# Is Phenylalanine Restricted Dietary Treatment in Phenylketonuria Associated with Disordered Eating: A Systematic Review

**DOI:** 10.3390/nu18152576

**Published:** 2026-08-06

**Authors:** Sharon Evans, Fatma Ilgaz, Anita MacDonald

**Affiliations:** 1 Birmingham Children’s Hospital, Steelhouse Lane, Birmingham B4 6NH, UK; anita.macdonald@nhs.net; 2 Department of Nutrition and Dietetics, Faculty of Health Sciences, Hacettepe University, Ankara 06100, Turkey; fatma.celik@hacettepe.edu.tr

**Keywords:** eating disorders, disordered eating, PKU, phenylketonuria

## Abstract

**Background**: In phenylketonuria (PKU), the lifelong phenylalanine (Phe)-restricted diet may contribute to altered feeding behaviours and disordered eating. The true prevalence of disordered eating and eating disorders remains uncertain, partly because validated PKU-specific assessment tools are lacking. This systematic review aimed to identify, appraise, and synthesise the evidence on disordered eating and eating disorders in PKU. **Methods**: Four electronic databases (PubMed, Scopus, Web of Science, and Cochrane) were searched from inception to 24 February 2026. English-language studies reporting disordered eating or eating disorders in early-diagnosed individuals with PKU managed with a Phe-restricted diet, with or without drug therapy, were included. Studies of late- or never-treated patients, pregnancy, lactation, reviews, and preclinical studies were excluded. Two independent reviewers conducted study selection and data extraction. Outcomes included the prevalence of eating disorders, disordered eating behaviours and attitudes, associated factors, and the influence of drug treatment. Findings were synthesised qualitatively, and risk of bias was assessed using the NIH Study Quality Assessment Tools. **Results**: Twenty-four studies (2928 individuals with PKU) met the inclusion criteria. Most were observational or survey-based. Eating disorders were reported more frequently in PKU (3–13%) than in the general population (*n* = 4 studies). Common disordered eating features included food neophobia (*n* = 5 studies), limited food variety (*n* = 9), food aversion or refusal (*n* = 5), poor appetite (*n* = 5), prolonged mealtimes (*n* = 3), negative parent–child mealtime interactions (*n* = 6), and specific taste preferences (*n* = 5). Delayed feeding skill development (*n* = 6 studies), reduced social eating (*n* = 6), gastrointestinal symptoms (*n* = 8), and psychological or neurodevelopmental difficulties (*n* = 7) were also associated with disordered eating. Early feeding difficulties frequently persisted into adulthood, and longstanding eating behaviours did not consistently improve with pharmaceutical treatment (*n* = 6 studies). Most studies were rated as fair quality (19/24), with common limitations including small sample sizes, lack of sample-size justification, non-validated assessment tools, and absence of randomised controlled trials. **Conclusions**: Current evidence suggests that people with PKU experience a greater burden of eating-related difficulties than the general population, although robust estimates of eating disorder prevalence remain lacking. Existing screening tools may misclassify treatment-related dietary behaviours as pathological or fail to identify PKU-specific eating concerns. Development of PKU-specific screening tools could support routine clinical discussions about eating behaviours and the psychosocial impact of dietary treatment, facilitating earlier identification of individuals requiring specialist eating disorder assessment. (Prospero registration: CRD42024539600).

## 1. Introduction

Phenylketonuria (PKU) is an inherited metabolic disorder caused by deficiency of the hepatic enzyme phenylalanine hydroxylase, resulting in elevated blood and brain phenylalanine (Phe) concentrations that, if untreated, lead to intellectual disability. Newborn screening and early initiation of a Phe-restricted diet within the first weeks of life have transformed outcomes, preventing severe neurocognitive impairment in most affected individuals [1]. Although pharmacological therapies, including sapropterin, sepiapterin, and pegvaliase, have expanded treatment options for selected patients [2,3,4,5,6,7,8], dietary management remains the principal treatment for most individuals with PKU.

Lifelong dietary treatment requires severe restriction of natural protein, supplementation with low-Phe protein substitutes, and adequate energy intake supported by special low-protein foods. For many individuals with classical PKU, natural protein tolerance remains below 10 g/day (approximately <500 mg/day Phe), representing only 10–20% of the protein intake consumed in an unrestricted diet [9]. Daily management requires continuous calculation of Phe intake, careful food selection, and avoidance of almost all high-protein foods. Natural protein intake is derived almost exclusively from plant foods, and many children grow up with little or no exposure to the taste, smell, and texture of animal-derived protein foods during critical periods of feeding [4,10,11,12,13,14,15,16,17,18,19].

These unique dietary experiences have the potential to shape food preferences and eating behaviours throughout life. Eating is an inherently multisensory process in which taste, smell, texture, appearance, and chemesthetic sensations interact to influence food acceptance, appetite, pleasure, and willingness to try unfamiliar foods [20,21]. In PKU, repeated exposure to strongly flavoured protein substitutes, combined with a limited repertoire of permitted foods, may alter sensory preferences, reduce the hedonic value of eating, and reinforce restricted eating behaviours. As a result, eating may become viewed primarily as a therapeutic necessity for maintaining metabolic control rather than a pleasurable or socially rewarding experience.

Disordered eating encompasses maladaptive eating attitudes and behaviours that do not fulfil diagnostic criteria for a recognised eating disorder but may nevertheless impair physical, psychological, or social functioning and increase the risk of developing a clinical eating disorder [22]. Eating disorders, including anorexia nervosa, bulimia nervosa, avoidant/restrictive food intake disorder (ARFID), and pica, are characterised by persistent disturbances in eating behaviour associated with significant medical or psychosocial consequences [22]. Whether the unique dietary demands of PKU contribute to the development of disordered eating or eating disorders remains poorly understood.

The dietary environment inherent to PKU management may create circumstances in which atypical eating behaviours develop, become reinforced, or are difficult to distinguish from treatment-related practices. From infancy, eating is highly structured, externally regulated, and continuously monitored. Meals are centred around prescribed protein allowances, repeated administration of low Phe protein substitutes, and restricted food choices, with little opportunity for spontaneous or flexible eating. Eating can become functional rather than pleasurable (sedative hunger), narrowing the sensory, emotional, or hedonic engagement with food [23] that normally accompanies flexible eating patterns. These experiences may contribute to food neophobia, food aversion, reduced appetite, fear of consuming prohibited foods, and avoidance of unfamiliar foods. Long-term dietary routines established during childhood may become deeply embedded and difficult to modify, even when greater dietary freedom becomes possible following pharmacological treatment.

Caregivers of children with PKU often experience considerable anxiety surrounding dietary adherence because poor metabolic control carries the risk of neurocognitive consequences. This may unintentionally promote controlling feeding practices, including excessive supervision, pressure to consume protein substitutes, or coercive mealtime behaviours, which may influence the development of healthy eating habits. Beyond the home, many individuals with PKU report eating separately from peers, feeling socially isolated, or experiencing embarrassment and bullying related to their dietary restrictions [24,25,26]. These experiences may further contribute to maladaptive eating behaviours and a conflicted relationship with food. Dietary patterns and feeding dynamics established in childhood frequently persist into adulthood, reinforcing an atypical and often conflicted relationship with food. Many adults with PKU choose to relax dietary restrictions, a pattern associated with increased anxiety and depression, reduced executive functioning, and attention deficits [27,28], all recognised risk factors for disordered eating.

Pharmacological treatments such as sapropterin and pegvaliase can substantially increase natural protein tolerance and improve opportunities for social eating and dietary variety in responsive individuals. Nevertheless, dietary liberalisation is not always straightforward. Long-established food preferences, sensory expectations, anxiety surrounding higher protein intake, and habitual eating behaviours may persist despite improved metabolic control, limiting the full psychosocial benefits of treatment.

Collectively, these factors suggest that individuals with PKU may be at increased risk of developing disordered eating behaviours. However, the true prevalence of disordered eating and eating disorders remains uncertain. Existing assessment tools were developed for the general population and may either misclassify treatment-related dietary behaviours as pathological or fail to identify PKU-specific patterns of eating-related difficulties. Consequently, clinically important problems may remain unrecognised and untreated.

This systematic review aimed to identify, critically appraise, and synthesise the available evidence relating to disordered eating and eating disorders in individuals with PKU. Specifically, we sought to: (1) determine the prevalence of disordered eating and eating disorders; (2) characterise the eating behaviours, attitudes, and feeding patterns associated with these conditions; (3) identify psychological, behavioural, and clinical factors associated with disordered eating; (4) examine factors contributing to their development and persistence; and (5) evaluate the influence of pharmacological therapies on eating behaviours. By synthesising the current evidence, this review aims to inform the development of validated PKU-specific assessment tools to support earlier recognition, routine clinical screening, and improved management of eating-related difficulties in this population.

## 2. Materials and Methods

This study was conducted using the Preferred Reporting Items for Systematic Reviews and Meta-Analyses (PRISMA) current guidelines [29]. It has been registered on PROSPERO: Disordered eating and eating disorders in phenylketonuria: a systematic review (CRD42024539600).

### 2.1. Literature Search

A systematic literature search was performed in 4 electronic databases (Medline/PubMed, Web of Science, Scopus, and the Cochrane Central Register of Controlled Trials) to identify articles, PhD theses, and conference abstracts published in English, with no date restriction. The final search was completed on 24 February 2026. Grey literature was identified through these database searches only. In addition, the reference lists of relevant narrative reviews, systematic reviews, and published guidelines were manually screened to identify any further eligible studies. No additional grey literature sources (e.g., institutional repositories or dedicated grey literature databases) were searched.

The search strategy included a combination of keywords that described the main condition (e.g., phenylketonuria OR PKU) and outcome (e.g., anorexia OR bulimia OR “binge eating” OR neophobia OR “eating disorder” OR “disordered eating”, etc.). The final search string is provided in Supplementary Table S1.

### 2.2. Study Selection Method

The PICO (population, intervention, comparison, and outcomes) framework was applied to formulate the review question. Eligible studies included original research such as observational designs (case–control, cohort, and cross-sectional studies), randomised controlled trials, and PhD theses or reports. Conference abstracts containing relevant data were also considered when full-text articles were unavailable. Preclinical studies (including in vitro and in vivo studies on cell cultures or animals), review articles, and studies lacking sufficient primary outcome data were excluded.

To be included, study populations were required to consist of patients with PKU at any age who met the following criteria: (1) early diagnosed and treated with a Phe-restricted diet, sapropterin, pegvaliase, or a combination of these therapies; or (2) early-treated individuals who discontinued treatment during adolescence or adulthood. Studies were excluded if they included patients who were late diagnosed or late treated (defined as treatment initiation after two months of age), never treated, or pregnant or lactating.

Screening was carried out manually by two independent reviewers (S.E. and F.I.). Inter-rater calculations were not conducted. Any disagreements were resolved through consensus or, when necessary, in consultation with a third reviewer (A.M.).

### 2.3. Outcome Measures and Data Extraction

Data were extracted independently by two authors (S.E. and F.I.) using a standardised Excel data extraction form. The following information was collected from all eligible studies: study characteristics (authors, publication year, country, setting, and study design); population characteristics (sample size, sex, age at diagnosis and treatment initiation, current age, PKU severity, ethnic origin, and PKU severity where available); treatment details (for diet-only treatment: degree of Phe restriction, methods of dietary intake assessment, and adherence; for pharmacological treatment: type of drug, dose, duration, adherence, and reported side effects); and study outcomes. Independent extraction ensured consistency and minimised bias in the synthesis of findings.

The primary outcomes included:

(1) types and patterns of eating disorders (e.g., anorexia nervosa, bulimia nervosa, binge eating disorder, pica, avoidant/restrictive food intake disorder) and disordered eating behaviours in PKU (e.g., food neophobia, orthorexia, restrictive eating, eating difficulties, food avoidance or refusal, poor appetite, irregular meals, food fads, food preoccupation, taste or oral aversion);

(2) the prevalence of eating disorders and disordered eating;

(3) the methods and instruments used to assess eating disorders or disordered eating;

(4) attitudes and behaviours associated with disordered eating (e.g., specific food choices, taste preferences, limited dietary variety, mealtime behaviour difficulties, parental stress at mealtimes, parental coercion or force-feeding, dieting behaviours, guilt related to consuming incorrect foods, self-harm, substance misuse);

(5) factors potentially contributing to disordered eating and eating disorders (e.g., mealtime environment, socioeconomic status, family structure, bullying, nutritional deficiencies); and

(6) any influence of pharmacological treatments (sapropterin or pegvaliase) on the presence, severity, or type of disordered eating behaviours.

Secondary outcome measures included anthropometric indicators (weight, height, and body mass index (BMI)) and metabolic control (blood Phe and Tyr concentrations) (see Supplementary Table S2).

Where necessary, corresponding authors of studies published within the past 10 years were contacted via email to obtain additional or clarifying information.

### 2.4. Data Analysis

Data from the included studies were synthesised qualitatively. Key findings were grouped into five main categories based on the primary outcomes: (1) “eating disorders” (including anorexia or bulimia nervosa, binge eating disorder and avoidant restrictive food intake disorder); (2) “disordered eating traits” (including weight/food preoccupation, self-esteem, body image, feeding difficulties and food aversion/refusal, selective eating, appetite, food/general neophobia); (3) “attitudes and behaviours potentially associated with disordered eating” (including mealtime behaviours and duration, caregiver mealtime behaviour/mood and coping strategies, mealtime stress and anxiety, taste and food preferences); (4) “factors potentially contributing to disordered eating” (including social and environmental eating restrictions, early feeding skills, gastrointestinal symptoms, and psychiatric and psychological disorders); (5) the same outcomes listed in 1–4 above following “pharmacological treatment” (including disordered eating, selective eating, food/general neophobia, eating behaviours, caregiver behaviours, social impact and psychiatric and psychological disorders). These categories were defined a priori and further refined during data extraction.

Secondary outcomes such as anthropometrics and metabolic control were summarised in descriptive tables using means ± SDs, medians, and range/IQRs as appropriate (see Supplementary Table S2)

### 2.5. Quality Appraisal and Risk of Bias Assessment Method

Two reviewers (S.E. and F.I.) independently assessed the methodological quality and the risk of bias of the included studies using the NIH (National Institutes of Health) Study Quality Assessment Tools (National Heart Lung and Blood Institute 2021). Depending on the study design, four tools were applied: the Quality Assessment of Controlled Intervention Studies, the Quality Assessment Tool for Observational Cohort and Cross-Sectional Studies, the Quality Assessment Tool for Before-After (Pre-Post) Studies With No Control Group, and the CASP checklist for Qualitative Research.

Each tool includes 10–14 items evaluating potential methodological limitations, including sources of bias (e.g., selection, performance, attrition, and detection), confounding, study power, and the strength of causal inference. Reviewers rated each item as “yes,” “no,” “cannot determine,” “not reported,” or “not applicable,” following the guidance provided for each tool.

Based on these ratings, studies were classified as “good” (low risk of bias), “fair” (some risk of bias but not sufficient to invalidate results), or “poor” (substantial risk of bias). Inter-rater calculations were not conducted. Any discrepancies between reviewers were resolved through discussion until consensus was reached.

## 3. Results

### 3.1. Study Selection

**Figure 1:**
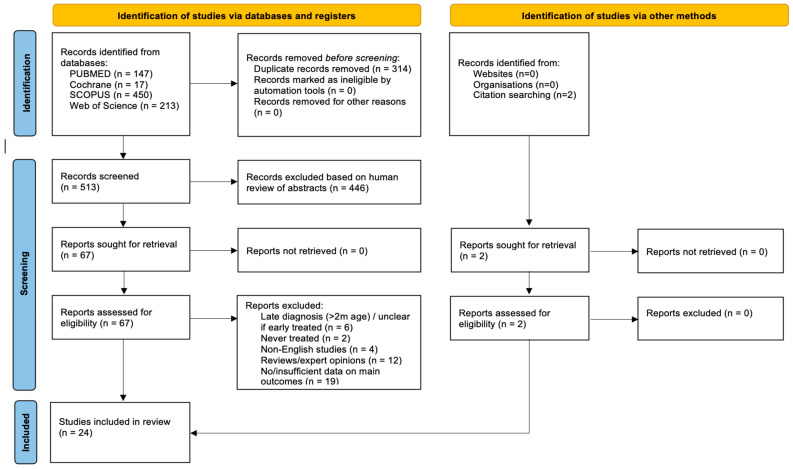
Study selection process according to PRISMA 2020 flow diagram (Page et al., 2021 [29]).

After removing duplicates, a total of 513 published studies and conference abstracts were identified from 4 electronic databases (Figure 1). Following title and abstract screening, 446 records were excluded, leaving 67 articles for full-text review. Of these, 24 studies met the inclusion criteria and were incorporated into the systematic review. Owing to the heterogeneity of outcome measures, assessment methods, and study designs, a meta-analysis was not undertaken.

### 3.2. Study Characteristics

Table 1 describes the main characteristics of included studies.

Across the included studies, the majority employed cross-sectional observational designs (*n* = 10), followed by prospective longitudinal observational (*n* = 4), retrospective cohort (*n* = 3), pre–post observational studies without control groups (*n* = 3), non-randomised controlled intervention studies (*n* = 2), qualitative cross-sectional research (*n* = 1), and one mixed-methods study combining cross-sectional and cohort elements. One cross-sectional observational study was available only as a conference abstract, although additional data were obtained directly from the authors [30]. Three studies [31,32,33] reported on the same cohort as earlier publications [34,35,36]; data for these studies were analysed once for each paired study, resulting in 21 unique datasets.

Studies were published between 1970 and 2026 and were conducted in the UK (*n* = 11), USA (*n* = 6), Germany (*n* = 2), and Australia, France, Ireland, the Netherlands, and Portugal (each *n* = 1). Collectively, the studies represent 2928 individuals with PKU (or 686 when excluding the large database study by Bilder [37]), with sample sizes ranging from 6 to 2242 (or 6 to 187 excluding Bilder [37]). Comparative healthy controls totalled 12,188 participants (or 207 excluding Bilder [37]).

Among studies reporting gender (*n* = 22/24), both sexes were represented (males *n* = 242; females *n* = 386). Ethnicity was infrequently reported (*n* = 7 studies), and most participants were White/Caucasian (*n* = 115/117). PKU severity was also inconsistently described (*n* = 9 studies); among those reporting severity+, 80% (*n* = 312/390) had classical PKU, 16.7% (*n* = 65/390) mild PKU, 2.6% (*n* = 10/390) hyperphenylalaninaemia, 0.5% (*n* = 2/390) dihydropteridine reductase deficiency, and 0.2% (*n* = 1/390) moderate PKU.

Participants were diagnosed through newborn screening or by ≤52 days of age, and were early-treated (≤14 days). One study [30] included a single late-diagnosed individual (3 years of age). Two studies did not report age at diagnosis; however, author correspondence confirmed that all participants were likely newborn-screened [38], and age and country context suggested newborn-screened cohorts in the remaining study [39]. These three studies were retained due to the presence of specific prevalence and outcome data on eating disorders or use of disordered eating assessment tools and the low likelihood or absence of late diagnoses based on feedback from authors.

Participant age at recruitment ranged from 3 months to 65 years. Most studies (63%, *n* = 15/24) focused exclusively on paediatric populations (≤17 years), 29% (*n* = 7/24) on adults (≥18 years), and 8% (*n* = 2/24) included adolescents and adults (≥11 years) [39,40].

**Table 1:** Main characteristics of included studies.

Reference	Country, Setting	Study Design	Group	N	Gender (M/F)	Ethnic Origin	PKU Severity	Age; Mean ± SD; *Median [Range]*		
								At Diagnosis (Days)	At Diet Initiation (Days)	During Study (m or y)
Bentovim et al., 1970 [41]	UK, Hospital (*n* = 1)	Prospective, non-randomised controlled intervention	PKU diet only: Group A: on protein hydrolysate	6	3:3	n/r	n/r	40.6 ± 22.4	n/r	3.8 ± 1.7 y
			PKU diet only: Group B: on L-AA	6	1:5			37.1 ± 21.7		4.1 ± 1.6 y
MacDonald et al., 1994 [34]; MacDonald et al., 1997 [31]	UK, Patient homes	Cross-sectional observational comparative	PKU diet only:	15	3:12	n/r	cPKU	n/r (NBS)	n/r (Early)	3 *[1–5] y*
			Healthy controls:	15	3:12	n/r	n/a	n/a	n/a	3 *[1–5] y*
Antisdel et al., 2000 [39]	USA, Summer camps (*n* = 2)	Cross-sectional observational comparative	PKU (treatment n/r):	30	0:30	n/r	n/r	n/r ^a^	n/r ^a^	19 ± 7.0 *[11–36] y*
			Type I Diabetes:	54	0:54				10 ± 2.0	16 ± 3.0 *[11–21] y*
			Healthy controls:	n/r	n/r		n/a	n/a	n/a	n/r
Thiele et al., 2015 [42]	Germany, Hospital clinic (*n* = 1)	Pre-Post retrospective observational	PKU diet only (Pre-BH4)	8	5:3	n/r	BH4 responsive	n/r (NBS)	n/r (Early)	10.5 ± 3.8 *[6.0–17]* y
			PKU BH4: (Post-BH4; fully liberalised diet)	5	5:3					At 3 y FU: 13.5 *[9–20] y*
			PKU diet + BH4: (Post-BH4; partially liberalised diet)	3						
			Healthy population:	n/r	n/r	n/r	n/a	n/a	n/a	Age matched
Evans et al., 2016 [35]; Evans et al., 2018 [32]	UK, Hospital (*n* = 1)	Non-randomised controlled intervention; cross-sectional observational comparative	PKU diet only:	35	17:18	n/r	n/r	<14	<14	*8.5* *[4–13] y*
			Healthy controls:	35	17:18	n/r	n/a	n/a	n/a	Aged-matched
Bilder et al., 2017 [37]	USA, Research database	Retrospective cohort	PKU diet only:	2242 ^b^	n/r	n/r	n/r	n/r (NBS)	n/r (Early)	25 ± 2.8 *[20–29] y* 34 ± 2.8 *[30–39] y*
			Diabetes Type I-II:	4334 ^c^	n/r		n/r	n/r	n/r	25 ± 2.8 *[20–29] y* 34 ± 2.8 *[30–39] y*
			General population:	11,981 ^c^	n/r		n/a	n/a	n/a	25 ± 2.8 *[20–29] y* 34 ± 2.9 *[30–39] y*
Roberts et al., 2017 [43]	Australia (n/r)	Qualitative cross- sectional interview (thematic analysis)	PKU diet only:	8 ^d^	0:8	n/r	n/r	n/r (soon after birth)	n/r (Early)	29.9 ± 7.9 *[21–42]* y
Evans et al., 2017 [44]	UK, Hospital (*n* = 1)	Retrospective cohort	PKU diet only:	31	16:15	n/r	cPKU (*n* = 29) mPKU (*n* = 2)	n/r (NBS)	n/r (Early)	*≤2 y*
Evans et al., 2019a [36]; Evans et al., 2019b [33]	UK, Hospitals (*n* = 3)	Prospective, longitudinal observational comparative	PKU diet only:	20	14:6	*n* = 20 Caucasian	n/r	*10* *[2–16]*	n/r (Early)	Weaning: *4.3 m* *[2.9–6.6 m]* At FU: *24 m*
			Healthy controls	20	12:8	*n* = 18 Caucasian *n* = 2 Caucasian/Afro-Caribbean	n/a	n/a	n/a	Weaning: *5.1 m* *[3.7–6.5 m]* At FU: *24 m*
Luu et al., 2020 [40]	USA, mail survey	Cross-sectional observational	PKU diet only:	12	9:6	Caucasian	n/r	n/r (NBS)	n/r (Early)	20.8 ± 7.1 *20 [12–34] y*
			PKU–Not on restricted diet:	3						
Viau et al., 2021 [45]	USA, Hospital (*n* = 1)	Cross-sectional observational	PKU pegvaliase only:	18	7:11	Caucasian	n/r	n/r (NBS)	n/r (Early)	38.2 ± 8.8 y
Coakley et al., 2022 [30]	USA, Online survey	Cross-sectional observational comparative	PKU (treatment n/r):	41	8:33	*n* = 40 Caucasian *n* = 1 Hispanic	n/r	n/r (*n* = 40 NBS *n* = 1 at 3 y) ^e^	n/r (*n* = 40 early)	31.2 y
			Healthy controls:	41	8:33	n/r	n/a	n/a	n/a	30.4 y
Haitjema et al., 2022 [38]	Netherlands, Online survey	Cross-sectional observational comparative	PKU diet only:	43	n/r	n/r	n/r	n/r ^g^	n/r ^f^	*7.0* *[1–16] y*
			PKU diet + BH4:	14						*7.5* *[2–16] y*
			Healthy controls:	50			n/a	n/a	n/a	*9.0* *[1–16] y*
Rice et al., 2023 [46]	Ireland, National IMD database	Mixed design: retrospective cohort, cross- sectional survey	PKU diet only:	39 ^g^	24:15	n/r	n/r	n/r (NBS)	8.3 ± 2.2	*≤13 m* (*n* = 39) 5.3 *[3.6–6.6] m* (*n* = 21 ^g^)
Rocha et al., 2023 [47]	Portugal, Online survey	Cross-sectional observational comparative	PKU diet only:	40	21:19	n/r	cPKU (*n* = 26) mPKU (*n* = 13) n/r (*n* = 1)	*23* *[IQR: 15–52]*	n/r (Early)	*2.0 y [7 m–6 y]* *[IQR: 2–4 y]*
			Healthy controls:	46	21:25	n/r	n/a	n/a	n/a	*3.5 y [7 m–6 y]* *[IQR: 2–5.3 y]*
Viau et al., 2023 [48]	USA, Metabolic clinics (*n* = 3)	Pre-Post prospective longitudinal	PKU pegvaliase only:	11 ^h^	4:7	Caucasian	n/r	n/r (NBS)	n/r (Early)	35 ± 13.3 y *[19.5–52.9 y]*
Pinto et al., 2025 [49]	UK, Hospital (*n* = 1)	Cross-sectional observational	PKU diet only:	30	16:17	n/r	cPKU (*n* = 19) mPKU (*n* = 13) n/r (*n* = 1)	n/r (NBS)	n/r (Early)	13.5 ± 1.3 y *[12–16 y]*
			PKU diet + BH4:	3						
Yilmaz et al., 2025 [50]	UK, Patient homes	Pre-Post prospective longitudinal	PKU diet only:	12	4:8	*n* = 11 Caucasian/European *n* = 1 Pakistani	cPKU (*n* = 11) moPKU (*n* = 1)	n/r (NBS)	n/r (Early)	*3.2 [IQR: 3.1–4] y*
Yilmaz Nas et al., 2026 [51]	UK, Hospital (*n* = 1)	Prospective longitudinal observational comparative	PKU diet only:	12	16:17	n/r	cPKU (*n* = 12)	n/r (NBS)	n/r (Early)	10.1 ± 4.2 y *[3–17 y]*
			PKU diet + BH4:	21			cPKU (*n* = 12) mPKU (*n* = 7) DHPR (*n* = 2)			10.4 ± 3.8 y *[3–17 y]*
Albers et al., 2026 [52]	Germany, Hospital (*n* = 1)	Retrospective cohort	PKU diet only:	36	n/r	n/r	n/r	n/r	n/r	n/r
			PKU BH4:	3		n/r	n/r	n/r	n/r	n/r
			PKU pegvaliase:	2		n/r	n/r	n/r	n/r	n/r
			Total:	41 ^i^	14:26 (n/r in *n* = 1)	n/r	cPKU (*n* = 36) mPKU (*n* = 4) mHPA (*n* = 1)	n/r (NBS)	n/r (Early)	32.8 ± 10.4 y ^j^ *[19–65 y]*
Giret et al., 2026 [53]	France, Hospital/IMD centre (*n* = 17)	Prospective longitudinal cohort	PKU diet only:	104	60:127	n/r	cPKU (*n* = 88) mPKU (*n* = 14) HPA (*n* = 2)	(NBS)	n/r (Early)	*27.2 y [22.3–32.3]*
			PKU off diet	83			cPKU (*n* = 62) mPKU (*n* = 10) HPA (*n* = 4)			
			(PKU BH4 ^k^):	(5)			cPKU (*n* = 2) mPKU (*n* = 3)			

Abbreviations; N: sample size; M/F: male/female; PKU: phenylketonuria; SD: standard deviation; m: months; y: years; L-AA: L-amino acid based protein substitute; n/r: not reported; n/a: not applicable; NBS: newborn screening; BH4: Sapropterin; FU: follow up; IQR: interquartile range; cPKU: classic phenylketonuria; mPKU: mild phenylketonuria; moPKU: moderate PKU; DHPR: dihydropteridine reductase deficiency; mHPA: mild hyperphenylalaninemia; IMD: Inherited metabolic disease. ^a^ Uncertain if any were late diagnosed but study included due to significance of data; all were born post NBS. ^b^ The combined subset of individuals aged 20–39 years was analysed to evaluate outcomes in an adult cohort who were most likely to have been diagnosed with PKU at birth through newborn screening, received early initiation of dietary treatment, and had a greater likelihood of benefiting from “early and continuous treatment” rather than only “early treatment”. Data on patients older than 39 years were not evaluated. ^c^ Only data on patients in the 20–39 year age group were evaluated for comparison. ^d^ *n* = 1 patient was off-diet, but all had normal IQ. ^e^ Study included despite 1 late-diagnosed patient due to significance of data and remaining patients all early diagnosed and treated. ^f^ Authors confirmed NBS. ^g^ Data on disordered eating during complementary feeding was only available for *n* = 21. ^h^ *n* = 6 were responders, and *n* = 5 were partial responders to Pegvaliase treatment. ^i^ Data were completed for only *n* = 36 patients; the remaining sample was lost-to-follow-up. Data on *n* = 4 patients who were late diagnosed were excluded from the analysis. ^j^ Data excludes 4 patients diagnosed late. Mean (SD) age of the *n* = 2 patients with anorexia nervosa diagnosis was: 21.9 6 ± 1.97 years. ^k^ Not clear if patients on BH4 were on a diet also.

### 3.3. Primary Outcomes

Table 2, Table 3, Table 4, Table 5 and Table 6 report primary outcome data.

#### 3.3.1. Eating Disorder Prevalence

Table 2 reports data from 5 included studies [30,37,43,52,53] on the prevalence of eating disorders in patients with PKU.

Across the PKU study population (*n* = 2519; range 8–2242), 2.8% (*n* = 70/2519) were reported to have an eating disorder, with individual study estimates ranging from 2.6% to 12.5%. Reported diagnoses included bulimia nervosa (*n* = 6/70), anorexia nervosa (*n* = 4/70), binge eating disorder (*n* = 1/70), and 59 cases of unspecified or undefined eating disorders. There is a suggestion that this may be more prevalent than in the normal population.

A large retrospective cohort study using a US insurance-claims database [37] did not specify the type of eating disorder but reported a 2.6% prevalence among individuals with PKU (*n* = 59/2242), compared with 0.6% in the general population (prevalence ratio = 4.7) and 1.6% in individuals with diabetes mellitus (prevalence ratio = 1.6). Four additional studies (2 cross-sectional [30,43], 1 retrospective cohort [52] and 1 prospective cohort [53]) reported prevalence based on confirmed diagnoses recorded in medical notes: bulimia nervosa (2.2%; *n* = 6/277), anorexia nervosa (1.4%; *n* = 4/277) and binge eating disorder (0.4%, *n* = 1/277); a total eating disorder prevalence of 4% (*n* = 11/277) across all four studies.

One study [30] used validated screening tools (SCOFF and BEDS-7) to assess eating-disorder prevalence in 41 adults with PKU compared with 41 matched controls. Although the prevalence of positive screening results for binge eating (38%; *n* = 16/41) and anorexia/bulimia nervosa (46%; *n* = 19/41) was higher in the PKU group, these differences were not statistically significant relative to controls.

Another study [53] highlighted that all patients in their cohort with an eating disorder (*n* = 5/152) had classic PKU, giving a prevalence of 3.3% within this PKU severity subgroup.

#### 3.3.2. Disordered Eating Traits

Table 3 reports data on disordered eating traits in patients with PKU from included studies.

Using eating disorder assessment tools validated for the general population, current studies suggest individuals with PKU may be at greater risk of disordered eating than the general population, particularly among those with poor metabolic control. The specific nature of disordered eating in PKU appears to differ from that characterising disordered eating in other chronic conditions such as diabetes. Feeding problems, limited food variety, poor appetite and food neophobia were all observed to be more prevalent in PKU than in the general population, although with the exception of food neophobia, validated data collection tools were rarely utilised.

Three observational cross-sectional studies [30,39,40] used standardised screening tools to estimate the possible prevalence of disordered eating in PKU. These included the Eating Attitudes Test (EAT-26) and the Eating Disorder Inventory (EDI/EDI-3). Although validated for use in the general population, none of these instruments has been validated for PKU, limiting the interpretability of scores in this context. One study [39] additionally administered the Coopersmith Self-Esteem Inventory (SEI) and the Body Cathexis Scale, which assess self-esteem and body image, respectively.

In one cross-sectional study using the EAT-26 [39], children with PKU scored higher than those with diabetes on the oral control subscale, indicating a perceived loss of self-control over eating and a greater sense of external pressure to gain weight. Conversely, they were less preoccupied with weight loss and avoiding high-fat foods than children with diabetes. Preoccupation with food and bulimic tendencies did not differ between groups. Overall, children with PKU demonstrated a higher risk of subclinical eating problems compared with normative data.

A second cross-sectional study [40] used an adapted version of the EAT-26 in adolescents and adults, comparing individuals on a restricted diet (*n* = 12) with those following an unrestricted diet (*n* = 3). Disordered eating was significantly more prevalent among participants with self-reported poor metabolic control than those adhering to a restricted diet. Reported concerns included preoccupation with food, particularly in the period preceding blood Phe monitoring, and worries about weight gain.

Using the Eating Disorder Inventory (EDI/EDI-3), individuals with PKU were found to differ significantly from normative data, indicating a heightened risk of developing subclinical eating problems [39]. Compared with individuals with diabetes, those with PKU were less preoccupied with weight, dieting, and binge/purging behaviours, but were more likely to endorse items reflecting a desire to retreat to the security of childhood, suggesting distinct psychological features within this population.

In a separate study using the EDI tool, poor metabolic control was significantly associated with higher BMI, greater body dissatisfaction, and increased psychological difficulties [30], further highlighting the interdependence of metabolic status, emotional wellbeing, and vulnerability to disordered eating in PKU.

In young children, feeding problems were consistently more prevalent than in the general population (*n* = 5 studies; 1 cross-sectional, 1 retrospective cohort, 1 mixed methods; *n* = 15–39 subjects), with food refusal, fussy eating, and self-induced vomiting frequently reported, particularly during the first 12 months of life [31,34,44,46]. There was one exception. A cross-sectional study using the Brazilian Infant Feeding Scale (EBAI) indicated no significant differences in feeding difficulties between children with PKU (*n* = 40) and healthy controls (*n* = 46) [47], suggesting that the magnitude of early feeding challenges may vary according to assessment method and population context.

Selective eating and a restricted variety of foods were consistently more evident in children with PKU than in their caregivers or healthy control children (*n* = 4 studies; 2 cross-sectional, 1 non-randomised controlled intervention, 1 prospective longitudinal; *n* = 8–43 subjects). This included a narrower range of accepted foods per meal, per day, or per week, as well as fewer between-meal snacks [31,32,33,34,35,38]. During periods of protein substitute transition, dietary variety was further reduced, particularly among children experiencing challenging transitions [50].

Children with PKU consumed significantly greater amounts of high-carbohydrate staple foods, such as low-protein bread, pasta, and cereals, than their caregivers or control children [32,33,35,42] (*n* = 3 studies; 1 non-randomised controlled intervention, 1 prospective longitudinal, 1 pre-post retrospective; *n* = 8–35 subjects). A substantial proportion of their intake comprised special low-protein foods (e.g., bread, pasta, milk substitutes) and fruit and vegetables, with minimal consumption of milk, dairy products, and foods of animal origin compared with control children [42].

Poor appetite was reported to be markedly more common in infants and children with PKU (*n* = 3 studies, 1 cross-sectional, 1 prospective longitudinal, 1 retrospective cohort; *n* = 15–31 subjects), affecting up to 53% (*n* = 8/15) of cases compared with 7% (*n* = 1/15) of control children [31,33,34,44]. This was attributed in part to the requirement for regular protein substitute administration, typically three times per day, which was described as suppressing appetite for food and contributing to reduced interest in eating [33].

Food neophobia was significantly more prevalent in children and adolescents with PKU than in the general population [35,38] (*n* = 2 studies, 1 non-randomised controlled intervention, 1 cross-sectional; *n* = 35–43 subjects). Importantly, this heightened neophobia did not appear to be associated with parental neophobia [32], suggesting that it may arise from PKU-specific dietary experiences rather than familial modelling. Children with PKU who were more food-neophobic also exhibited a markedly more restricted dietary variety [50]. Among adolescents, females were more likely than males to display food neophobia [49].

#### 3.3.3. Attitudes and Behaviours Potentially Associated with Disordered Eating

Table 4 reports attitudes and behaviours potentially associated with disordered eating.

Utilising validated assessment tools, there are suggestions that mealtime behaviour, child–parent mealtime interactions, meal duration and parental anxiety are often more negatively affected in children with PKU than in non-PKU children.

Negative mealtime behaviours were more common in young children with PKU than in healthy controls (*n* = 3 studies; 2 cross-sectional studies utilising validated assessment tools, 1 retrospective cohort; *n* = 15–43 subjects), including self-induced vomiting or gagging, spitting out food, crying, and tantrums [31,34,38,44]. Mealtime enjoyment was notably lower among children with PKU [36,38] (*n* = 2 studies; 1 prospective longitudinal, 1 cross-sectional; *n* = 20–43 subjects), and meal duration was significantly longer, with children feeding more slowly and requiring greater parental prompting [31,34,38] (*n* = 2 cross-sectional studies, 1 utilising a validated assessment tool (*n* = 15 subjects). In contrast, one study using a non-validated questionnaire and clinical records in very young children reported no differences in mealtime stress, distractions, or feeding duration between PKU and control groups [47]. Nevertheless, broader evidence indicates that prolonged mealtimes may persist into adolescence based on a cross-sectional study (*n* = 15 subjects) utilising the validated EAT-26 assessment tool [40].

Caregiver stress, anxiety, and reduced mealtime enjoyment were often more pronounced in PKU than in the general population (*n* = 3 studies; 1 prospective longitudinal, 1 cross-sectional, 1 mixed methods; *n* = 2 studies utilising validated assessment tools; *n* = 20–43 subjects), particularly during the weaning period and most notably between 12 and 18 months of age. This period often coincided with rising blood Phe levels, teething, intercurrent illness, and the child’s emerging independence, all of which contributed to heightened parental strain [36,38,46]. In a mixed-design study, up to 52% (*n* = 11/21) of parents self-reported significant challenges, and 82% (*n* = 17/21) reported feeling stressed during weaning, driven by food refusal, fussy eating, maintaining target blood Phe, calculating protein content, administering protein substitute, educating extended family, avoiding forbidden foods, and managing meals outside the home [46]. However, one earlier cross-sectional study reported no differences in mealtime stress between mothers of children with PKU and control mothers [31], highlighting potential variation related to study era and measurement tools.

Compared with parents of healthy children, caregivers of children with PKU in 2 cross-sectional studies using non-validated tools (*n* = 15–43 subjects) demonstrated less verbal interaction during meals [31,34], adopted stricter responses to food spillage, and reported greater frustration and anger [38]. They also described more difficulty offering a varied diet, less confidence that their child had eaten enough, and a greater tendency to offer alternative foods when the child refused what was served [38]. A more rigid parental feeding approach was significantly associated with poorer metabolic control [38].

Children with PKU have been reported to show distinct sensory and food-related preferences compared with non-PKU children across 3 studies (2 non-randomised controlled interventions, 1 cross-sectional; *n* = 6–35 subjects), including a dislike of sweet foods [31,34], a preference for savoury foods (e.g., vegetables) [35], and often strongly flavoured items such as pickled onions, curry sauce, and olives [31,34,41]. However, early and repeated exposure to bitter-tasting protein substitutes does not appear to shape or alter taste preferences in PKU [32,35].

#### 3.3.4. Factors Potentially Contributing to Disordered Eating

Table 5 reports on factors potentially contributing to disordered eating in patients with PKU.

Whilst most studies described parent/patient reported data or data from non-validated assessment tools, there was evidence from several studies suggesting delayed development of feeding skills during weaning in PKU compared to control children. There was also a suggestion that the dietary restrictions of PKU may impact on the social aspects of eating, including eating meals as a family and eating outside of the home. Gastrointestinal and psychiatric/psychological symptoms, particularly anxiety and depression, also appear to be more common in PKU than in the general population. However, the correlation between these factors and disordered eating remains to be explored in PKU.

Early feeding skills in children with PKU differed from those of healthy children in several important respects [31,33,34,44,47], and these early differences could potentially increase vulnerability to disordered eating (*n* = 4 studies, 2 cross-sectional, 1 prospective longitudinal, 1 retrospective cohort; *n* = 15–31 subjects). Reported challenges include prolonged bottle-feeding during weaning (*n* = 3), delayed introduction of textured foods (*n* = 1), delayed self-feeding, and extended periods of parental spoon-feeding (*n* = 2) [31,33,34,44,47]. Together, these patterns suggest a trajectory of slower progression through key feeding milestones, which may shape later eating behaviours and mealtime dynamics in PKU.

The social dimensions of eating may also influence vulnerability to disordered eating in PKU. Children with PKU were more likely to eat meals alone at home, rather than with the rest of the family, compared with non-PKU children [31,33,34,38] (*n* = 3 studies, 2 cross-sectional, 1 prospective longitudinal; *n* = 15–43 subjects). In a qualitative cross-sectional study of *n* = 8 adults, as children they reported feeling different or isolated from peers during social eating occasions; in adolescence, this often manifested as frustration, reduced dietary adherence, and avoidance of shared meals; whilst as adults they continued to describe long-standing difficulties eating in social contexts, leading to avoidance of dining out [43]. Conversely, positive parental support, including consistent inclusion in family meals and active facilitation of food-related skills, was associated with better psychological outcomes in adulthood, fewer social eating difficulties, and improved dietary adherence [43].

Gastrointestinal (GI) symptoms, including vomiting, burping, retching, abdominal pain, flatulence, constipation, and diarrhoea, were more common in individuals with PKU than in the general population [31,33,34,44] (*n* = 3 studies, 1 cross-sectional, 1 prospective longitudinal, 1 retrospective cohort; *n* = 15–31 subjects). Using the Brazilian Infant Feeding Scale (EBAI), Rocha et al. [47] found that children (*n* = 40) with greater feeding difficulties also experienced significantly more GI symptoms, highlighting the close interplay between GI discomfort and problematic feeding behaviours.

In adults with PKU, a prospective longitudinal study (*n* = 41) and a retrospective cohort study (*n* = 2242) identified that the prevalence of several physical and neurological disorders was higher than in the general population, including sleep disturbance, pain disorders, migraine and headache, fatigue and malaise, movement disorders/tremors, epilepsy and convulsions, multiple sclerosis, and dementia [37,52]. Adults with PKU who have co-existing psychiatric disorders were also more likely to report stomach aches [52], suggesting that somatic symptoms may cluster with psychological distress and potentially influence eating behaviour.

Psychological and psychiatric disorders, both of which are closely linked to disordered eating, are reported to be more common in individuals with PKU than in the general population [37,43,49] (*n* = 3 studies, 2 cross-sectional, 1 retrospective cohort; *n* = 8–2242 subjects). In childhood, this may manifest as tantrums, anxiety, clinginess, and obsessive behaviours [41]. Children with poor psychological adjustment to their condition were significantly more likely to exhibit symptoms of disordered eating in a cross-sectional study (*n* = 30) [39].

In another cross-sectional study of adolescence, assessments using the validated Hospital Anxiety and Depression Scale (HADS) identified abnormal anxiety in 40% (*n* = 10/25) and abnormal depression in 12% (*n* = 3/25) of participants [49]. Using the validated EuroQol-5D-5L, 4% (*n* = 1/25) reported extreme, 16% (*n* = 4/25) severe, and 20% (*n* = 5/25) moderate anxiety and depression. Additionally, 15% (*n* = 5/33) reported suicidal or self-harm thoughts, and 12% (*n* = 4/33) had a diagnosis of autism or ADHD [49]. Adolescent females with PKU were twice as likely as males to experience anxiety and depression [49].

In adulthood, a retrospective cohort study (*n* = 2242) demonstrated an increased prevalence of psychiatric conditions in PKU compared with both the general population and individuals with diabetes, including autism, ADHD, and obsessive–compulsive disorder [37]. Most other psychiatric disorders also occurred more frequently in adults with PKU than in the general population, encompassing anxiety, depression, personality disorders, phobias, behaviour and conduct disorders, psychosis/schizophrenia, Tourette syndrome, and intellectual disabilities [37].

Two studies (1 prospective longitudinal, 1 retrospective cohort) reported 26% (*n* = 48/187) and 27% (*n* = 11/41) of individuals with PKU as having at least one psychiatric condition [52,53] and 15% (*n* = 6/41) had multiple diagnoses [52]. PKU severity or being “on-“ or “off-diet” did not appear to influence the overall incidence of psychiatric comorbidities, although those with classic PKU were twice as likely to have depressive episodes compared to those with moderate PKU [53]. Individuals with psychiatric comorbidity were significantly more likely to exhibit higher blood Phe levels, fewer results within the target range, and lower dietary adherence [52]. Among adults with PKU, the greatest impact on quality of life was attributed to the practical burden of protein restriction and the feelings of guilt associated with lapses in dietary adherence [52]. Collectively, these findings highlight the substantial emotional and behavioural pressures inherent in lifelong dietary management and their potential to exacerbate vulnerability to disordered eating.

#### 3.3.5. Traits, Attitudes, Behaviours and Other Factors Potentially Contributing to Disordered Eating in Patients on Pharmacological Treatment

Table 6 reports the traits, attitudes, behaviours and other factors potentially contributing to disordered eating in patients on pharmacological treatment.

Evidence from studies evaluating pharmacological therapies for PKU suggests that dietary liberalisation may be accompanied by improvements in selected eating behaviours. Pegvaliase treatment has been associated with reductions in uncontrolled and emotional eating, while sapropterin has been shown to increase dietary variety. However, consumption of high-protein foods generally remains lower than in healthy controls and is partly dependent on the degree of improvement in natural protein tolerance. Studies using validated assessment tools have also reported reductions in food neophobia and improvements in the social aspects of eating following treatment with both sapropterin and pegvaliase. Despite these positive findings, caregiver anxiety and depression appear to remain largely unchanged, and the effects of pharmacological therapies on broader psychological and psychiatric outcomes in individuals with PKU remain largely unexplored.

In a pre-post prospective longitudinal study (*n* = 11), the Three-Factor Eating Questionnaire Revised-18 (TFEQ-R18) was used to assess disordered-eating risk. Uncontrolled eating (i.e., difficulty stopping eating or feeling out of control around food) and emotional eating (i.e., eating in response to stress, sadness, anxiety, or emotions rather than hunger) were found to decrease significantly in individuals with PKU who benefited from pegvaliase, a pharmacological treatment that improves protein tolerance. These improvements were observed after 15 months of treatment and were more pronounced than in individuals who experienced only partial benefit [48].

Food selectivity and limited dietary variety have been shown to improve with sapropterin treatment in 2 studies using unvalidated assessment tools (1 pre-post retrospective, 1 prospective longitudinal), with children consuming a broader range of protein-rich foods [42,51], including regular bread, pasta, rice, dairy products, and meat. In one study (*n* = 8), after two years of treatment, intake of breads, cereals, meat, fruit, vegetables, fats, and sweets was comparable to that of healthy peers, although consumption of dairy products and fish remained lower; conversely, children with PKU consumed twice as much potato, pasta, and rice [42]. In another study, after two years of sapropterin, responders (*n* = 21/33) showed significant increases in intake of cow’s milk, regular cheese, meat, fish, eggs, regular bread, and regular pasta, with greater dietary variety strongly associated with higher natural-protein tolerance [51].

Food neophobia in PKU has also been shown to improve with both sapropterin [51] (*n* = 1 prospective longitudinal study; *n* = 33 subjects), and pegvaliase treatment [45,48] (*n* = 2 studies, 1 pre-post prospective longitudinal, 1 cross-sectional; *n* = 11–18 subjects). Adults receiving pegvaliase reported greater enjoyment of food and were less fearful of trying new foods, reflecting a change in eating-related confidence and flexibility [45,48]. However, some degree of neophobia may persist; in one study, 18% (*n* = 3/17) of pegvaliase-treated individuals continued to experience high levels of food neophobia [45].

Using the Hospital Anxiety and Depression Scale (HADS), caregivers of children receiving sapropterin in a prospective longitudinal study (*n* = 33) showed a significant improvement in depressive symptoms, but no corresponding improvement in anxiety, over two years of treatment. However, differences between responders and non-responders were not significant, suggesting that any psychological benefit for caregivers may be modest [51]. Similarly, in a cross-sectional study (*n* = 14) using the behavioural paediatrics feeding assessment scale (BPFAS), caregiver feelings about mealtimes were unchanged, and those with responsive children still felt that their child’s eating pattern might have a negative influence on their child’s health, more so than caregivers of control children [38].

The social impact of the PKU diet also appears to improve with both sapropterin [51] (*n* = 14 subjects) and pegvaliase treatment [48] (*n* = 11 subjects). For children, sapropterin enabled more frequent consumption of meals outside the home and facilitated greater social participation, including activities such as sleepovers [51]. Among adults, pegvaliase responders in a prospective longitudinal study (*n* = 11) demonstrated increased enjoyment of eating and enhanced engagement with the social aspects of meals, as assessed using the Epicurean Eating Tendencies Scale. These improvements were closely associated with the removal of dietary protein restrictions, which allowed individuals to participate more fully and spontaneously in shared eating experiences [48].

Improvements in psychological and psychiatric disorders associated with pharmacological treatment in PKU have largely not been demonstrated. However, one study reported a significant reduction in adolescent anxiety after two years of sapropterin treatment [51].

#### 3.3.6. Summary of Factors Associated with Disordered Eating

Table 7 provides a summary of factors that may contribute to, or protect against, the development of disordered eating.

### 3.4. Quality Appraisal and Risk of Bias Assessment

**Figure 2:**
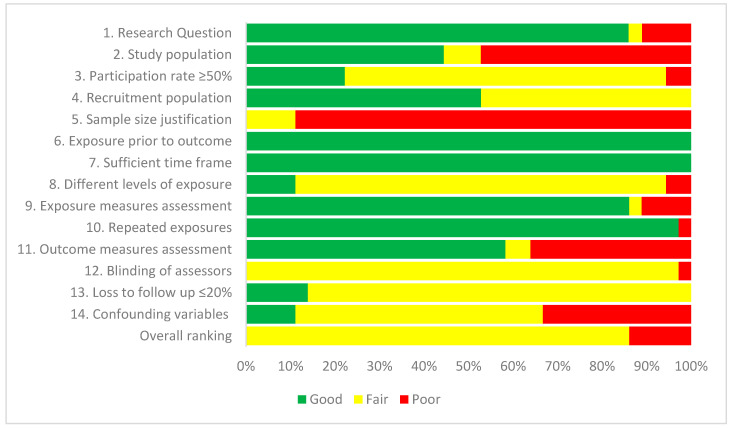
Quality appraisal and risk of bias of observational cohort and cross-sectional studies (*n* = 18).

**Figure 3:**
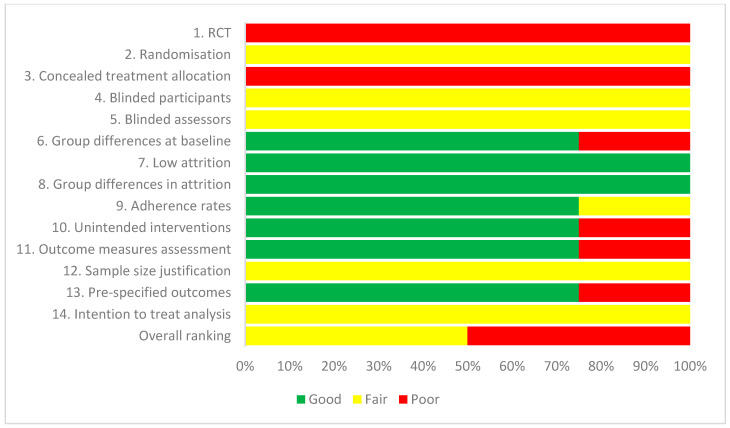
Quality appraisal and risk of bias of controlled intervention studies (*n* = 2).

**Figure 4:**
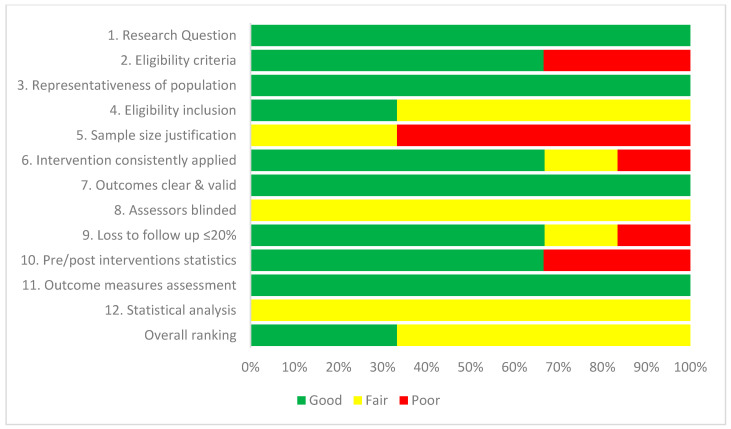
Quality appraisal and risk of bias of Before-After (Pre-Post) studies with no control group (*n* = 3).

**Figure 5:**
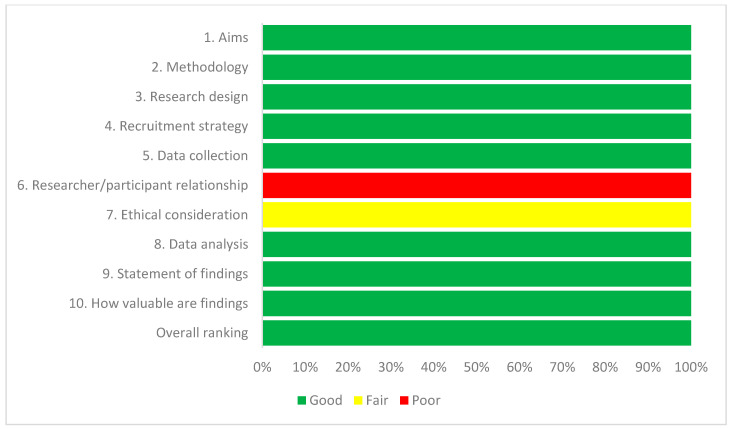
Quality appraisal and risk of bias of qualitative research studies (*n* = 1).

A summary of the risk of bias assessment is shown in Figure 2, Figure 3, Figure 4 and Figure 5. Using the NIH Quality Assessment Tools, most studies were rated as “fair” (*n* = 19) with only *n* = 3 studies rated as “poor” and *n* = 2 as “good”. For observational cross-sectional and cohort studies (*n* = 18) [30,31,32,33,34,36,37,38,39,40,44,45,46,47,49,51,52,53], common limitations included poor descriptions of the study population, absence of sample-size justification, use of non-validated assessment tools, and inadequate statistical control for confounding variables. Participation rates and loss-to-follow-up were frequently not reported or unclear, and several items relating to different levels of exposure were often not applicable to the study designs.

For the controlled intervention studies (*n* = 2) [35,41], the main issues were the absence of randomised controlled trials, lack of randomisation, and no treatment concealment or blinding. In one study, baseline group differences were not fully controlled, interventions varied both within and between groups, and the study lacked a clearly articulated hypothesis, instead presenting exploratory suggestions. Sample-size justification and intention-to-treat analysis were also unclear or absent.

For the before–after (pre–post) studies without a control group (*n* = 3) [42,48,50], key concerns included lack of sample-size justification, unclear eligibility criteria, and statistical analyses based on small samples with insufficient power to detect meaningful differences. It was often unclear whether all eligible participants were enrolled, whether assessors were blinded, and several criteria relating to the use of individual-level data to infer group-level effects were not applicable.

For the single qualitative study [43], the primary limitation was the lack of consideration of the researcher–participant relationship, alongside insufficient clarity regarding ethical issues.

## 4. Discussion

This systematic review is the first to collectively and systematically evaluate disordered eating and eating disorders in individuals with PKU. Following PRISMA guidelines, the review covers literature up to 24 February 2026. Most of the 24 included studies were observational (cross-sectional, cohort, or case–control), and five were survey-based. Only five papers reported directly on eating-disorder prevalence [30,37,43,52,53] or used validated (non-PKU-specific) eating-disorder assessment tools [39,40]. Consequently, much of the available literature focuses on disordered eating rather than formally diagnosed eating disorders.

The existing but limited evidence suggests that medically diagnosed eating disorders may be more common in adults with PKU than in other long-term conditions, such as diabetes, and compared with the general population [37,54,55,56,57]; with reported prevalence rates of between 2.6 and 12.5%. Bilder et al. [37] found that 2.6% of adults aged 20–39 years with PKU (59/2242) had a documented eating-disorder diagnosis, compared with 0.6% in the general population, while Albers et al. [52] reported a prevalence of 5% in their PKU cohort. This increased prevalence may reflect the pressure of lifelong dietary restriction, combined with the condition-related impact on mood, impulse control, and emotional regulation, particularly in individuals with elevated blood Phe levels [1,27,58,59,60,61]. Conversely, regular monitoring of adults with PKU in specialist metabolic clinics may facilitate more consistent detection and documentation of eating disorders than in the general population, potentially contributing to higher reported rates.

In the general population, eating disorders and disordered eating are typically more prevalent in females, although increasing rates are now recognised among males [62,63]. Existing tools for assessing eating-disorder risk are not designed to capture the unique nuances of the PKU diet, which may limit their interpretive accuracy in this population. Nevertheless, using the validated EAT-26 and Eating Disorder Inventory (EDI), Antisdel et al. [39] identified 23% of women with PKU as “symptomatic” for disordered eating, particularly in relation to self-control around food, perceived pressure to eat, and maturity fears (i.e., a desire to retreat to the security of childhood). This proportion was lower than that observed in women with diabetes mellitus (33%), who demonstrated greater weight-related preoccupation. Symptomatic women with PKU showed poorer psychological adjustment and lower self-esteem than non-symptomatic women, although anthropometric measures did not differ [39]. The authors concluded that females with PKU differ from normative data on both EAT-26 and EDI, suggesting an increased vulnerability to subclinical eating problems.

There was evidence that individuals with poor metabolic control report greater concerns about weight gain and engage more frequently in weight-loss behaviours [40], with high blood Phe associated with higher BMI and increased body dissatisfaction [30,64], and over half of adults with PKU expressing concerns about their weight [65]. While these findings suggest an interaction between metabolic status, body image, and eating-related attitudes [66], the literature remains limited by small samples, self-reported measures, and a lack of PKU-specific tools capable of distinguishing normative body-image concerns from clinically significant disordered eating. Comparisons with other chronic conditions requiring dietary management, such as cystic fibrosis, coeliac disease, IBD, and diabetes, highlight that disordered eating behaviours and maladaptive mealtime environments are well documented across medically complex populations [39,67,68,69,70,71,72,73]. Importantly, in these conditions the chronic illness almost always precedes the onset of disordered eating [68], a directionality that is highly plausible in PKU given that dietary treatment begins in infancy. However, current PKU research rarely interrogates the mechanisms linking metabolic instability, psychological distress, and weight-related behaviours, nor does it adequately consider the role of treatment burden, stigma, or limited food choice.

In this systematic review, evidence indicated that food aversion and refusal of food or protein substitute started in the weaning period and, for some, persisted into later childhood and adulthood [31,34,41,44,46]. Delayed weaning onto solids and postponing the introduction of second-stage protein substitutes were associated with greater food refusal, fussy eating, delayed self-feeding, and prolonged bottle use [31,33,34,44,46,47], and both early and late weaning (<17 weeks or >26 weeks) have been linked to increased feeding difficulties [33,36,44]. From the collective studies, individuals with PKU were often described as less interested in food and showing more negative mealtime behaviours than their peers [31,34,35,36,41,44]. However, most studies relied on small samples, parent-reported outcomes, and inconsistent definitions of “negative behaviour,” making it difficult to determine if food-related tantrums, avoidance of high-protein foods, or vomiting reflect feeding pathology or predictable responses to a medically imposed, highly restrictive diet. Therefore, from the data, it is difficult to disentangle the effects of developmental feeding challenges, stress-driven parent–child dynamics, and adaptive dietary learning. In the future, progress requires PKU-specific, validated behavioural tools and longitudinal study designs capable of identifying causal pathways

Meal duration was typically longer in children with PKU, with 67% taking more than 20 min to complete a meal compared with 20% of control children [31,34,40,47]. Several studies reported that children with PKU were less likely to sit at the table with other family members, often eating meals at a different time [33,34,38]. However, some of this reflects treatment-driven logistics rather than intrinsic child factors. The literature rarely accounts for the structural barriers that make shared family meals, known to be protective against disordered eating and obesity, more challenging for families managing PKU [38]. Caregiver stress when eating outside the home further illustrates how the social organisation of meals is shaped by treatment burden, not solely by child behaviour.

Social eating challenges extended into adolescence and adulthood, with limited availability of suitable foods leading to avoidance of dining out [43]. From reported studies, individuals with PKU frequently described feeling different from peers in settings such as school, restaurants, or social gatherings, often perceiving PKU as a burden and describing feelings of isolation or distress, particularly when questioned about their diet in public [43]. Overall, there is a lack of validated measures of social eating burden in PKU, meaning that constructs such as “feeling different” or “social avoidance” remain poorly operationalised and inconsistently interpreted. As a result, current conclusions risk over-attributing psychosocial distress to individual vulnerability rather than recognising the role of inaccessible food environments and societal misunderstanding of medical diets.

Across the studies, there is a consistent pattern that individuals with PKU rely on a narrow, repetitive dietary repertoire and show heightened reluctance to try unfamiliar foods. Studies report that many children and adults consume the same limited foods day after day and, in some cases, fewer meals or snacks than peers [31,32,33,34,36,38,44,64,65,74]. Food neophobia is significantly more common in PKU than in age-matched controls and is closely linked to reduced dietary variety [32,35,38,45,48,50,66,74,75]. Adults similarly describe anxiety around unfamiliar foods and reliance on a restricted set of “safe” options [25,37,45,74,76], with some evidence of developmental and gender-related differences in vulnerability [49,74]. While these patterns are often interpreted as evidence of reduced dietary variety or appetite, the literature rarely interrogates the treatment-driven constraints that shape these behaviours, including the limited availability and palatability of special low-protein foods and the appetite-suppressing effects of protein substitutes taken multiple times per day [31,33,34,36,41,44]. A more robust evidence base will require objective dietary-intake assessments, PKU-specific behavioural instruments, and ecologically grounded research designs that capture how treatment burden and food availability shape everyday eating patterns.

Parents of children with PKU consistently report higher anxiety, frustration, and stress during mealtimes than parents of children without PKU, with the 12–18-month period described as particularly challenging due to weaning difficulties and increasing blood Phe levels [34,36,38,46]. Increased prompting around both food and protein substitute intake that escalates to coercion or force-feeding, may create an aversive mealtime environment. This may potentially disrupt a child’s appetite self-regulation and independent feeding development [31,33,34,36,47,68,77]. Parents also engage in less verbal interaction during meals, show lower confidence in their child’s intake, prepare alternative foods when items are rejected, and enforce stricter rules around food spillage [31,34,38]. Rigid parental approaches have been associated with poorer metabolic control, while inconsistent routines contribute to difficulties during transitions between protein substitutes [38,50]. In contrast, supportive practices, such as including the child in family meals and actively fostering self-feeding skills, were linked to more favourable long-term outcomes, with adults reporting fewer social-eating difficulties and greater confidence in managing their diet in social contexts if childhood mealtimes were positive and inclusive [43]. However, the published evidence rarely distinguished between maladaptive parental responses and the structural pressures of managing a medically restricted diet, nor does it examine how clinical messaging, fear of metabolic instability, or the emotional weight of dietary responsibility shaped parental behaviour in a structured way. Future research should prioritise observational studies of real-world mealtimes, longitudinal designs to track how parental anxiety evolves across developmental stages, and PKU-specific measures of parental feeding behaviour to identify modifiable targets for intervention and support.

Individuals with PKU also show distinct taste preferences compared with controls. Early studies suggested a preference for savoury, strongly flavoured foods and a relative dislike of sweet foods [31,34,41], although these early patterns likely reflect immature sensory development and exposure to bitter-tasting protein substitutes. As children grow and sweetened protein substitutes become more common, this “bitter taste imprinting” appears to decline [32,35,78]. Despite these shifts, individuals with PKU consistently rate vegetables more favourably than peers [32,35,38]. However, the long-term implications of these preferences for eating behaviour or risk of disordered eating remain unclear. Future work should use controlled sensory testing, developmentally sensitive longitudinal studies, and PKU-specific taste-preference tools to clarify how early dietary exposures shape later food choices and eating patterns.

GI symptoms such as constipation, diarrhoea, vomiting, and abdominal pain appear more common in individuals with PKU than in the general population [25,31,33,34,47,64]. Similarly to other GI conditions, these symptoms co-occur with feeding difficulties including reduced dietary variety, food refusal, and gagging [68]. Given the well-established comorbidity between GI symptoms and disordered eating, and evidence from other diet-restricted conditions that disordered eating typically follows the primary diagnosis, GI discomfort in PKU may contribute to or exacerbate maladaptive eating behaviours. However, the extent of this influence on the development or maintenance of disordered eating remains unclear.

Psychological and neurodevelopmental conditions, including ASD, ADHD, anxiety, depression, and obsessive–compulsive traits, occur at higher rates in PKU than in diabetes or the general population [37,49,79,80], and each can influence appetite regulation, sensory processing, mealtime behaviour, and dietary flexibility. This systematic review suggested that within the context of a highly restrictive therapeutic diet, these vulnerabilities amplified feeding challenges [38,52,64]. Children with PKU who exhibit psycho-behavioural difficulties were more likely to show problematic feeding patterns such as food refusal, heightened neophobia, and rigid eating routines [81], while neurodevelopmental conditions like ASD and ADHD may compound difficulties due to sensory sensitivities and aversion to food textures. Anxiety and obsessive–compulsive traits may further intensify preoccupation with food rules or fear of dietary mistakes, particularly around protein substitute intake.

Studies examining feeding behaviour during pharmacological treatment for PKU show that entrenched eating patterns often persist despite increased dietary flexibility [19,38,42,45,48,51,82]. Many individuals simply increase portion sizes of familiar high-carbohydrate foods while continuing to avoid protein-rich options, raising the risk of micronutrient deficiencies when natural protein intake rises without adequate protein-substitute use [42,45]. More than half of adults on pegvaliase show persistent food neophobia and low food enjoyment, with limited improvement even when protein intake is liberalised [45]. Sapropterin-related improvements in neophobia appear restricted to those with higher protein tolerance [51]. Across therapies, social aspects of eating improve more reliably than core eating behaviours [38,48,51]. Overall, the evidence indicates that pharmacotherapy alone does not modify long-standing avoidance, sensory aversions, or restricted dietary repertoires, highlighting the need for integrated behavioural and nutritional interventions to address persistent maladaptive eating patterns.

Overall, disordered eating in adulthood may reflect the cumulative effects of early feeding challenges, rigid dietary routines, negative food experiences, and the psycho-social pressures of lifelong dietary management. Difficulties commonly reported by adults with PKU, such as weight gain, food-related conflict, poor psychological health, and restricted or repetitive eating patterns, mirror established correlates of disordered eating. Qualitative accounts from individuals with PKU describe an unpleasant or conflicted relationship with food, including the use of food as a reward or a source of emotional regulation [25]. Even adults who are no longer following a low-protein diet frequently continue to avoid protein-containing foods, often unintentionally [83], and many remain highly food-neophobic [74]. Although recent adult-care consensus guidelines [84,85] acknowledge the need to identify disordered eating, the suggested screening questions are unvalidated for PKU and risk missing the atypical, treatment-driven behaviours characteristic of this population [57]. The absence of validated tools represents a significant gap: without PKU-specific screening instruments, early detection is inconsistent, misclassification is likely, and opportunities for timely intervention are lost. Developing and validating PKU-specific assessment measures is therefore essential to improve clinical recognition, guide appropriate referral pathways, and support targeted interventions across adulthood.

This systematic review highlights substantial gaps in the evidence base surrounding feeding difficulties and disordered eating in PKU. Across studies, there is no consensus definition of disordered eating that reflects the unique dietary demands of PKU, making it difficult to distinguish treatment-driven behaviours, such as rigid food rules, preoccupation with intake, or avoidance of high-protein foods, from clinically significant psychopathology. An important limitation of the current evidence base is that almost all studies relied on eating disorder screening instruments developed and validated for the general population rather than for individuals with PKU. Consequently, these tools may misclassify treatment-related dietary practices, such as meticulous food weighing, protein calculation, dietary restraint, or preoccupation with food, as pathological behaviours, while simultaneously failing to identify PKU-specific manifestations of disordered eating. This limitation substantially restricts interpretation of the reported prevalence estimates and reinforces the need to develop and validate assessment tools specifically designed for the unique dietary and psychosocial context of PKU.

Furthermore, longitudinal data are scarce, leaving the developmental progression from early feeding problems to adolescent and adult eating-disorder risk poorly understood. Few studies integrate the influence of neuro-developmental or psychological comorbidities, despite their higher prevalence and likely contribution to maladaptive eating. Evidence on how pharmacological treatments reshape eating behaviour is limited, short-term, and based on small samples, and qualitative accounts of lived experience remain rare. No intervention trials have evaluated strategies to prevent or treat disordered eating in PKU. Collectively, these findings demonstrate an important need for PKU-specific conceptual frameworks, validated assessment tools, and longitudinal, multidisciplinary research to advance understanding and improve care across the lifespan

The available literature on disordered eating in PKU is sparse, heterogeneous, and methodologically inconsistent, limiting the strength of conclusions that can be drawn. Dietary intake variables (energy, Phe, natural protein, protein equivalent from protein substitutes, and total protein) and nutritional or biochemical biomarkers were not reported due to inconsistent and infrequent reporting. Most studies use small samples, lack control groups, and rely on inconsistent and non-validated measures that do not account for the unique dietary demands of PKU, increasing the risk of misclassification and under- or over-estimation of disordered eating. For this reason, two studies that did not fully meet the inclusion criteria (because they may have contained small numbers of late-diagnosed or late-treated patients) were still included because they provided valuable outcome data relevant to the review. Cross-sectional designs dominate the field, preventing inferences about causality or developmental tracking from early feeding difficulties to later eating-disorder risk. Reporting of key variables, such as metabolic control, treatment modality, neurodevelopmental comorbidities, and psychosocial context, is inconsistent, making comparisons across studies difficult. Evidence on the impact of pharmacological treatments on eating behaviour remains limited and short-term. Finally, qualitative perspectives and lived-experience data are scarce, restricting understanding of how individuals with PKU perceive and navigate their relationship with food. These limitations highlight the need for robust, longitudinal, and PKU-specific research to advance the field.

## 5. Conclusions

Current evidence suggests that individuals with PKU experience a greater burden of eating-related difficulties than the general population; however, the true prevalence of clinically diagnosed eating disorders remains uncertain because of the limited quality of the available evidence and the absence of validated PKU-specific assessment tools. Future prospective longitudinal studies using robust diagnostic criteria, larger sample sizes and condition-specific assessment instruments are needed to distinguish adaptive treatment-related behaviours from clinically significant disordered eating and to support earlier identification and appropriate intervention.

## Figures and Tables

**Table 2:** Data from included studies on the prevalence of eating disorders in patients with PKU.

Reference	Sample (Population vs. Comparator)	Types of Eating Disorder	Method of Assessment or Diagnosis	Prevalence
Bilder et al., 2017 [37]	Adults with PKU (*n* = 2242) vs. Diabetes (Type I/II; *n* = 4334) vs. General population (*n* = 11,981);	Eating disorders (general, not defined)	**ICD-9 codes on database claims;** Prevalence ratio (PR)	**PR for eating disorders:** PKU: 2.6% (*n* = 59/2242) vs. Diabetes: 1.6% (*n* = 71/4334) vs. general population: 0.6% (*n* = 67/11,981) PKU/Diabetes ratio: 1.6 (1.15–2.24; *p* < 0.05) PKU/General population ratio: 4.7 (3.32–6.64; *p* < 0.05) **Overall–**Significantly higher in adults with PKU compared with adults with Diabetes and the general population
Roberts et al., 2017 [43]	Adults with PKU on diet (*n* = 8)	Bulimia nervosa Other psychiatric disorders	**Patient medical history**	Major depressive disorder and recurrent bulimia nervosa 12.5% (*n* = 1/8)
Coakley et al., 2022 [30]	Adults with PKU (*n* = 41) vs. Healthy controls (*n* = 41)	Eating disorder (Anorexia and bulimia nervosa)	**SCOFF questionnaire:** 5 questions; screening tool for anorexia and bulimia nervosa	At study commencement 7% (*n* = 3/41) had a diagnosed ED Existing diagnosis: anorexia nervosa *n* = 1, bulimia nervosa *n* = 1 **Screening positive on SCOFF–**adults with PKU vs controls 46% (*n* = 19) vs. 34% (*n* = 14); NS
		Binge eating	**Binge Eating Disorder Screener-7 (BEDS-7):** 7-item; screening tool for binge eating	Existing diagnosis: BED *n* = 1 **Screening positive on BEDS-7—**adults with PKU vs controls 38% (*n* = 16) vs. 34% (*n* = 14); NS
Albers et al., 2026 [52]	Adults with PKU on diet (*n* = 36), on BH4 treatment (*n* = 3) and on pegvaliase treatments (*n* = *2*)	Anorexia nervosa	**Patient records of confirmed diagnoses**	4.9% (*n* = 2/41)
Giret et al., 2026 [53]	*n* = 187 adults with PKU: on diet (*n* = 104), off diet (*n* = 83), on BH4 treatment (*n* = 5) ^a^	Anorexia nervosa Bulimia nervosa	**Medical records and interviews**	Total eating disorders: 2.7% (*n*=5/187) ^b^ Anorexia nervosa: 0.5% (*n* = 1) ^b^ Bulimia nervosa: 2.1% (*n* = 4) ^b^ Total cPKU with eating disorders: 3.3% (*n* = 5/152) ^b^

Abbreviations: PKU: phenylketonuria; ICD: International Classification of Diseases; PR: prevalence ratio; ED: eating disorder; SCOFF: The Sick, Control, One, Fat, Food Questionnaire; BEDS-7: Binge Eating Disorder Screener-7; NS: not statistically significant. ^a^ Not clear if patients on BH4 were on diet also. ^b^ Unclear if these included patients “on” or “off diet” or on BH4 treatment; however, the authors report that an analysis of the prevalence of psychiatric disorders in patients “on diet” and “off diet” did not find any statistically significant difference (*p* = 0.11).

**Table 3:** Data on disordered eating traits in patients with PKU from included studies.

Reference	Sample (Population vs. Comparator)	Outcomes	Method of Assessment	Key Findings
A. **Disordered eating assessment tools:** Outcomes related to risk of eating disorders				
Antisdel et al., 2000 [39]	Children with PKU (*n* = 30) vs. Children with Type I diabetes (*n* = 54) vs. Healthy children (n/r) (all females)	Weight/food preoccupation, food avoidance, binge eating/purging, eating self-control	**Eating Attitudes Test-26 (EAT-26) (validated) ^a^:** 26-item; 6-point Likert scale (“never” 0 to “always” 5); higher scores = greater symptomatology; scores ≥ 20 were classified as “symptomatic”	**EAT-26 ^a^:** **Total (symptomatic):** No significant difference in problematic eating between PKU and diabetes (23% vs. 33%) **Diet subscale ^a^:** PKU scored significantly lower than diabetes **Oral control subscale ^a^:** PKU scored significantly higher than diabetes **Bulimia subscale ^a^:** No significant difference between PKU and diabetes **PKU vs. healthy children:** Higher risk of developing subclinical eating problems compared to normative data **Overall:** PKU symptomatic participants demonstrated poorer psychological adjustment and lower self-esteem than non-symptomatic peers. Symptoms of disordered eating were not as severe as those of patients with eating disorders, but females with PKU reported more intense symptoms than those reported by nonclinical samples.
		Weight/food preoccupation, binge eating/purging, maturity fears	**Eating Disorder Inventory (EDI) (validated) ^b^:** 64-item; 8 subscales; higher scores = greater symptomatology	**EDI ^b^:** **Drive for Thinness subscale ^b^:** PKU scored significantly lower than diabetes **Bulimia subscale ^b^:** PKU scored significantly lower than diabetes (after correction for age) **Maturity Fears subscale ^b^:** PKU scored significantly higher than diabetes **Overall:** PKU differed from normative data (higher risk of developing subclinical eating problems)
		Self-esteem	**Coopersmith Self-esteem Inventory (SEI) (validated):** Rated as “like me” or “unlike me”; higher scores = higher levels of self-esteem	**Self-esteem:** Significantly lower in patients who had symptoms of disordered eating (both PKU and diabetes) compared with non-symptomatic
		Body image	**Body Cathexis Scale (validated):** Evaluation of feelings about 45 characteristics of the body; 5-point Likert scale (“strong negative” 1 to “strong positive” 5); Higher scores = more positive body image	**Body image scores:** No significant difference between symptomatic patients vs. non-symptomatic patients with PKU; More negative body image in diabetes patients who had symptoms of disordered eating
Luu et al., 2020 [40]	Adolescents and adults with PKU (*n* = 15; *n* = 3 not on restricted diet)	Weight/food preoccupation, food avoidance, binge eating/purging, eating self-control	**Adapted version of Eating Attitudes Test (EAT-26) ^c^:** 38 questions, 6-point Likert scale (“always” to “never”); Lower scores = Symptomatic for behaviour; responses compared “restricted-diet” vs “unrestricted diet”	**Disordered eating behaviours (e.g., binge eating and intentional restriction):** significantly more frequent in children with self-reported poor metabolic control vs. good metabolic control (7/38 vs. 1/38 of questions) Patients on “restricted diet” were significantly more likely to report disordered eating traits for 2/38 items compared to those on “unrestricted diet” **Weight concerns: i**ndividuals who felt they did not manage their PKU well were more worried about gaining weight and engaged in activities to lose weight more often. **Preoccupation with food:** greater in individuals adhering to a protein-restricted diet, particularly in the period preceding blood Phe monitoring
Coakley et al., 2022 [30]	Adults with PKU (*n* = 41) vs. Healthy controls (*n* = 41)	Weight/food preoccupation, binge eating/purging, body image, insecurity	**Eating Disorders Inventory-3 (EDI-3) (validated) ^d^:** 91 questions; 6-choice format; self-reported; measures psychological features of eating disorders	**EDI-3 scores ** **^d^** **:** **Drive for thinness, Bulimia, Body dissatisfaction, Overall eating disorder risk:** no significant difference between groups **Ineffectiveness, Interpersonal problems, Overcontrol, General psychological maladjustment:** significantly lower in PKU than controls **Poor metabolic control:** significantly correlated with higher BMI and body dissatisfaction, and psychological problems (ineffectiveness, affective problems, and general psychological maladjustment)
B. **Feeding problems or difficulties in children**: Outcomes related to food refusal or aversion				
MacDonald et al., 1994 [34]; MacDonald et al., 1997 [31]	Children with PKU (*n* = 15) vs. Healthy children (*n* = 15)	Feeding problems	**Feeding assessment questionnaire (validated):** 5-point Likert scale; parent-reported	**Overall (at least one):** significantly higher in PKU vs. controls (93% vs. 33%; *p* < 0.02) **Difficult to feed:** significantly higher score for PKU vs. control (2.8 vs. 1.4; *p* < 0.0001)
Evans et al., 2017 [44]	Infants with PKU (*n* = 31)	Food refusal or acceptance (during weaning and transition to weaning PS)	**Patient dietetic records from weaning age until 2 y**	**Food refusal (child well):** 61% (on >1 occasion in 26%) **Food refusal (child unwell):** 52% (on >1 occasion in 32%) **Food refusal during teething:** 45% (on >1 occasion in 26%) **Self-induced vomiting with food:** 6% (on >1 occasion in 3%) Teething and intercurrent illness negatively affected acceptance of solid foods **Overall:** refusal of food was more predominant than PS refusal up to 12 months of age, after which the latter is more predominant. Timely introduction, gradual progression to more textured foods, and consistent feeding routines improved successful acceptance of solids
Rocha et al., 2023 [47]	Children with PKU (*n* = 40) vs. Healthy children (*n* = 46)	Feeding difficulties	**EBAI (Brazilian Infant Feeding Scale):** 14 items; 7-point Likert scale; Scores 61–65: Mild difficulties, 66–70: Moderate difficulties, >70: Serious difficulties	**Total EBAI score: **similar between infants with PKU and controls (54 [IQR: 45.25–60.75] vs. 50 [IQR: 46–61]; *p* = 0.808) **Frequency of feeding difficulties:** similar between infants with PKU and controls (25% vs. 28.3%; *p* = 0.810) **Mild—**PKU vs. controls (12.5% vs. 13%; NS) **Moderate—**PKU vs. controls (2.5% vs. 8.7%; NS) **Serious—**PKU vs. controls (10% vs. 6.5%; NS) No significant correlation between EBAI score and PKU severity
Rice et al., 2023 [46]	Children with PKU (*n* = 39)	Food refusal or acceptance	**Survey questionnaire (non-validated), medical notes**	**Challenges during transition to complementary feeding:** 52% of parents reported challenges including food refusal and fussy eating
C. **Limited variety/Selective eating:** Outcomes related to dietary variety and meal frequency				
MacDonald et al., 1994 [34]; MacDonald et al., 1997 [31]	Children with PKU (*n* = 15) vs. Healthy children (*n* = 15)	Selective eating; meal frequency	**3-day food diary**	**Range of foods and portions:** significantly less in PKU vs. control (21 foods vs. 36 foods over 3 days) **Foods offered per meal:** significantly fewer in PKU vs. control (meal: 2 vs. 4) **Number of snacks:** significantly fewer in PKU vs. control (1 vs. 2.6) **Eating a limited variety of foods:** no significant difference between groups (PKU: 67% vs. Control: 27%); mean score: PKU 3.1 vs. Control: 1.5 (*p* < 0.03)
Thiele et al., 2015 [42]	Children with PKU (*n* = 8) diet only vs. Healthy population	Food intake and dietary variety	**3-day food diary**	**% of total food consumption—PKU diet only vs. healthy German controls:** SLPFs—39% vs. 0% Milk and dairy—3% vs. 23% Bread, cereals—1% vs. 9% Food of animal origin (meat, egg, fish)—<1% vs. 9% **Overall—**patients with PKU allocated a substantial proportion of their total food intake to fruit, vegetables and SLPFs (e.g., low protein bread, pasta and milk substitutes)
Evans et al., 2016 [35]; Evans et al., 2018 [32]	Children with PKU and their parents (*n* = 35) vs. Healthy children and their parents (*n* = 35)	Selective eating	**FFQ (unvalidated):** 60-item tailored for PKU **Feeding Development Diary ^e^** (non-validated, qualitative): 3-day diary; parent-reported; Assessments: monthly to 12 m, then at 15, 18 and 24 m of age	**Variety of foods:** Significantly less in children with PKU than controls (median no. of foods/week: 33 vs. 54); and both parents (PKU mothers: 40; PKU fathers: 44) No significant difference in food variety between control children (median no. of foods/week: 54) and their parents (Control mothers: 51; control fathers: 52) **Food intake and food preference:** Children with PKU consumed significantly more sweets, sweetened drinks, sweet biscuits (LP), pasta (LP), strawberry, sweet potato and crisps than controls Intakes of fruit and vegetables and most other foods were similar across PKU and control groups Children with PKU ate significantly more high-carbohydrate staple foods such as LP bread, pasta and snack foods such as LP sweet biscuits and cake than parents Both groups of children ate significantly more sweets, sweetened drinks, ice-cream and potato fries than parents The differences between parent and child were greater for children with PKU **Number of daily meals/snacks:** one less meal/snack of solid food per day in PKU compared to controls
Evans et al., 2019a [36]; Evans et al., 2019b [33]	Infants with PKU (*n* = 20) vs. Healthy controls (*n* = 20)	Meal frequency	**Feeding Development Diary ^e^ (non-validated, qualitative):** 3-day diary; parent-reported; Assessments: monthly to 12 m, then at 15, 18 and 24 m of age	Children with PKU: consumed one less meal/snack of solid food per day compared to controls during weaning; and often consumed an additional liquid meal (Phe-free formula) compared to controls
Haitjema et al., 2022 [38]	Caregivers of: Children with PKU diet only (*n* = 43) vs. Healthy children (*n* = 50)	Dietary variety	**PKU-specific questionnaire:** 18 questions; parent-reported; to assess PKU-specific social restrictions and eating problems; 5-point Likert scale (“never” 1 to “always” 5 and “not stressful” 1 to “very stressful” 5)	Caregivers of children with PKU reported more difficulty in offering food variety compared to caregivers of control children (score 3 vs. 2; *p* < 0.001)
Yilmaz et al., 2025 [50]	Children with PKU (*n* = 12)	Dietary variety	**Food frequency questionnaire (FFQ) (validated):** 89-item; validated for PKU	**Median (IQR) number of different foods/week:** smooth transition (*n* = 5) vs. challenging transition (*n* = 7). Final: 38 (31–44) vs. 28 (26–31) (*p* = 0.05) **Overall**—Median number of different foods eaten/week was higher in the smooth transition group compared to the challenging transition group at all assessment periods, particularly the final visit (*p* < 0.05)
D. **Poor appetite:** Outcomes related to appetite and low food intake				
MacDonald et al., 1994 [34]; MacDonald et al., 1997 [31]	Children with PKU (*n* = 15) vs. Healthy children (*n* = 15)	Poor appetite	**Feeding assessment questionnaire (validated):** 5-point Likert scale; parent-reported	**Poor appetite:** significantly more common in PKU vs. controls (53% vs. 7%); mean score: PKU: 3.8 vs. Control: 2.7 (*p* < 0.01) **Low food intake:** significantly higher score for PKU vs. control (3 vs. 1)
Evans et al., 2017 [44]	Infants with PKU (*n* = 31)	Poor appetite	**Patient medical records from weaning age until 2 y**	**Poor appetite:** 23% (*n* = 7) of caregivers described their child as having poor appetite (on >1 occasion in 10%)
Evans et al., 2019a [36]; Evans et al., 2019b [33]	Infants with PKU (*n* = 20) vs. Healthy controls (*n* = 20)	Appetite	**Feeding Development Diary ^e^ (non-validated, qualitative):** 3-day diary; parent-reported; Assessments: monthly to 12 m, then at 15, 18 and 24 m of age	Children with PKU were less likely to be offered snacks between meals in case this decreased appetite for Phe-free weaning PS
E. **Food and general neophobia:** Outcomes related to reluctance to eat new or different foods				
Evans et al., 2016 [35]; Evans et al., 2018 [32]	Children with PKU and their parents (*n* = 35) vs. Healthy children and their parents (*n* = 35)	Food and general neophobia	**Neophobia scale (validated):** 9 food and 5 general questions; 7-item Likert scale (“always” to “never”); parent-reported	**General neophobia: c**hildren with PKU were significantly more neophobic than controls (total score: 18.8 vs. 22.1; *p* = 0.05), and their parents; Control children exhibited similar neophobia to their mothers, but were significantly more neophobic than their fathers **Food neophobia:** children with PKU were significantly more food neophobic than controls (total score: 34.5 vs. 46.2; *p* = 0.0003); Children in both groups were significantly more food neophobic than their parents. There was a limited influence of parental attitudes on children’s eating behaviour Children with PKU were significantly more likely than controls to be particular about what foods they eat and were less trusting and more fearful of new foods Neophobia did not make children less social because both groups were equally comfortable talking with or sitting next to a stranger
Haitjema et al., 2022 [38]	Caregivers of: Children with PKU on diet (*n* = 43) vs. Healthy children (*n* = 50)	Food neophobia	**PKU-specific questionnaire:** 18 questions; parent-reported; to assess social restrictions and eating problems; 5-point Likert scale (“never” 1 to “always” 5; “not stressful” 1 to “very stressful” 5)	**Difficulty trying new foods:** children with PKU on dietary treatment only were less likely to try new foods compared with healthy controls (*p* < 0.05)
Yilmaz et al., 2025 [50]	Children with PKU (*n* = 12)	Food and general neophobia (during transition from second to third-stage PS)	**Food Neophobia Scale (FNS; validated):** 14-item: 9 items related to food and 5 to general neophobia; 7-point Likert scale (“always” 1 to “never” 7); lower scores = more neophobia	Positive correlation between the number of different foods/week consumed by children and food neophobia score at each assessment (r > 0.6; *p* < 0.05), suggesting that children who were more neophobic (lower food neophobia scores) had significantly less food variety in their diets
Pinto et al., 2025 [49]	Adolescents with PKU (*n* = 25)	Food and general neophobia	**The Food Neophobia Scale (FNS); (validated):** 10-item self-report instrument; 7-point Likert scale (“always” 1 to “never” 7); higher scores = greater neophobia	**Food neophobia scores (mean ± SD scores):** **Afraid to eat things that never had before:** 4 ± 2.1 (Males: 5.3 ± 1.8 vs. Females: 3.1 ± 1.8; *p* = 0.009) **Overall**—The cohort did not exhibit strong indicators of food neophobia Females demonstrated significantly higher neophobic behaviour compared to males (mean scores: 3.5 vs. 4.1; *p* = 0.012) **General neophobia scores (mean ± SD scores)** **:** Avoid speaking to unknown people: 3.1 ± 2.1 (Males: 4.0 ± 2.2 vs. Females: 2.5 ± 2.0; *p* = 0.044) Don’t like sitting next to unknown people: 3.7 ± 2.5 (Males: 4.7 ± 2.2 vs. Females: 3.1 ± 2.5; *p* = 0.04)

Abbreviations: PKU: phenylketonuria; EAT-26: Eating Attitudes Test-26; EDI: Eating Disorder Inventory; PS: protein substitute; m: months; EBAI: Brazilian Infant Feeding Scale; IQR: interquartile range; FFQ: food frequency questionnaire; BMI: body mass index; FNS: Food Neophobia Scale; Phe: phenylalanine; SD: standard deviation. ^a^ **Eating Attitudes Test (EAT-26) subscales:** (1) Diet subscale—avoidance of fattening foods and preoccupation with weight loss; (2) Bulimia and Food Preoccupation subscale—food preoccupation and bulimic behaviour; (3) Oral Control subscale—self-control of eating and perceived pressure from others to gain weight. ^b^ **Eating Disorder Inventory (EDI) subscales:** (1) Drive for Thinness subscale—concerns with dieting, preoccupation with weight, and fear of weight gain; (2) Bulimia subscale—binge eating and purging; (3) Body Dissatisfaction subscale—negative body image and dissatisfaction with body shape; (4) Ineffectiveness subscale—feelings of inadequacy, insecurity, worthlessness, emptiness, and lack of control over one’s life; (5) Perfectionism subscale—achievement drives; (6) Interpersonal Distrust subscale—feelings of alienation and reluctance to form close interpersonal relationships; (7) Interoceptive Awareness subscale—uncertainty in identifying emotional states and visceral sensations related to hunger and satiety; (8) Maturity Fears subscale—desire to retreat to the security of childhood. ^c^ Some questions were eliminated because they would be answered abnormally by individuals with PKU. Additional questions were added to elucidate the cause of behaviour that might otherwise be considered “disordered” in the general population. ^d^ **Eating Disorders Inventory-3 (EDI-3) subscales:** (1) Drive for thinness; (2) Bulimia subscale; (3) Body dissatisfaction; (4) Low self-esteem; (5) Personal alienation; (6) Interpersonal insecurity; (7) Interpersonal alienation; (8) Interoceptive deficits; (9) Emotional dysregulation; (10) Perfectionism; (11) Ascetism; (12) Maturity fear. ^e^ **Feeding Development Diary**: to assess stage of texture progression, feeding skills, feeding environment, gastrointestinal symptoms, and behavioural difficulties administering weaning PS.

**Table 4:** Data on attitudes and behaviours potentially associated with disordered eating in patients with PKU from included studies.

Reference	Sample (Population vs. Comparator)	Outcomes	Method of Assessment	Key Findings
A. **Mealtime behaviour:** Outcomes related to eating behaviour and emotions				
MacDonald et al., 1994 [34]; MacDonald et al., 1997 [31]	Children with PKU (*n* = 15) vs. Healthy children (*n* = 15)	Negative feeding behaviour, verbal interactions, and meal duration	**Mealtime positive-negative rating scale:** 5-point Likert scale **Video of a midday meal**	**Negative feeding behaviours:** **Overall incidence:** similar between groups (PKU 41 vs. control 29) **Push food away:** PKU 47% vs. control 53% **Spit food:** PKU 47% vs control 27% **Chews/not swallow:** PKU 20% vs. control 40% **Turns head away:** PKU 40% vs. control 7% **Closes mouth:** PKU 27% vs. control 33% **Cries/screams:** PKU 20% vs. control 0% **Dribbles food:** PKU 7% vs. control 0% **Vomiting and gagging:** significantly greater in the PKU group (*p* < 0.04) **Need for parental prompting to begin eating:** significantly more common in PKU vs. control (67% vs. 7%) **Duration of meals:** significantly longer in PKU vs. control (24 min vs. 16 min) **Prolonged meals (>20 min):** significantly more common in PKU vs. control (60% vs. 20%); in 28% of PKU children, it took >30 min **Slow to feed: s**ignificantly more common in PKU vs. controls (67% vs. 20%) **Number of mouthful/min:** significantly less in PKU vs. control (60% of PKU ate < 2/min vs. 100% of control children ate >2/min)
Evans et al., 2017 [44]	Infants with PKU (*n* = 31)	Mealtime behaviour	**Patient medical records from weaning age until 2 y**	6% **self-induced vomiting** with food (3% on >1 occasion)
Evans et al., 2019a [36]; Evans et al., 2019b [33]	Infants with PKU (*n* = 20) vs. Healthy controls (*n* = 20)	Mealtime enjoyment	**Mealtime emotion (non-validated):** 5 statements, 5-point Likert scale; parent-reported; Assessments: at weaning, 8, 12, 15, 18 & 24 m of age	**Mealtime enjoyment:** less for children with PKU vs. controls between 8 and 24 months; children with PKU were reported to be significantly “less happy at mealtimes” over time than their control peers
Luu et al., 2020 [40]	Adolescents and adults with PKU (*n* = 15; *n* = 3 not on restricted diet)	Meal duration	**Adapted version of Eating Attitudes Test (EAT-26) ^a^:** 38 questions, 6-point Likert scale (“always” to “never”); Lower scores = symptomatic for behaviour;	**Meal duration:** time required to complete meals was abnormally prolonged in adolescents with PKU
Haitjema et al., 2022 [38]	Caregivers of: Children with PKU on BH4 (*n* = 14) vs. Healthy children (*n* = 50)	Mealtime behaviour and distractions	**Behavioural Paediatrics Feeding Assessment Scale (BPFAS; validated) ^b^:** 35 items: 25 items on child behaviour and 10 items on parent feelings around mealtime behaviour; 5-point Likert scale (“never” 1 to “always” 5; parent-reported. Higher scores = more problems	**Children with PKU on diet alone vs. healthy controls:** Total BPFAS child behaviour (median, range): 50 [35–76] vs. 45 [30–84]; *p* = 0.012 **PKU total sample:** During mealtime, younger children (1–7 y) were reported to get up from the table more often than older children (8–16 y) (*p* < 0.05) Younger children (1–7 y) more often whined or cried and had tantrums during mealtime than older children (8–16 y) (*p* < 0.05)
Rocha et al., 2023 [47]	Children with PKU (*n* = 40) vs. Healthy children (*n* = 46)	Mealtime stress and distractions	**Questionnaire (non-validated), clinical records**	**Stress during feeding:** similar between infants with PKU vs. controls (18% vs. 28%) **Distractions during mealtime (TV, tablet, phone, etc.):** similar between infants with PKU vs. controls (0–22.5% vs. 0–32.6%; *p* = 0.329) **Duration of feeding:** similar between infants with PKU vs. controls: <20 min: 43% vs. 30% 20–30 min: 50% vs. 50% >30 min: 8% vs. 20%
**B. Caregiver Factors:** Emotional and Psychological Aspects; Practices and Coping Strategies at Mealtimes				
MacDonald et al., 1994 [34]; MacDonald et al., 1997 [31]	Children with PKU (*n* = 15) vs. Healthy children (*n* = 15)	Mealtime stress and parental strategies	**Mealtime positive-negative rating scale:** 5-point Likert scale **Video of a midday meal**	**Mealtime stress:** no significant difference between groups as reported by mothers **Verbal interaction (e.g., asking if the food is nice) with parents during meals:** significantly less in PKU vs. control (5.5 times/10 min vs 12.5 times) **Use of parental strategies to persuade child to eat:** no significant differences between groups
Evans et al., 2019a [36]; Evans et al., 2019b [33]	Infants with PKU (*n* = 20) vs. Healthy controls (*n* = 20)	Parental anxiety and coping strategies	**Mealtime emotion (non-validated):** 5 statements, 5-point Likert scale; parent-reported; Assessments: at weaning, 8, 12, 15, 18 & 24 m of age **Coping Health Inventory for Parents (CHIP) (validated) ^c^:** (Score 0 “not helpful” to 3 “extremely helpful”); self-reported (mothers); Assessments: At weaning, 8, 12, 15, 18 & 24 m **Beck’s anxiety (validated):** 21-item multiple choice self-report (“not at all” to “severe”); self-reported (mothers) Assessments: At weaning, 8, 12, 15, 18 & 24 m	**Maternal scores in anxiety for emotive/cognitive symptoms:** peaked when the child was 15 m old **Mealtime enjoyment:** less for parents of children with PKU vs. controls between 8 and 24 m 12–18 m was identified as a key period when mothers feel most anxious/stressed with feeding, coinciding with raised blood Phe levels probably associated with teething, illness and developing independence **Link with metabolic control:** Mean blood Phe levels were highest and more variable between 13 and 18 m of age (*p* = 0.04) in line with levels of maternal stress (significant correlation at 15 and 18 m) and levels of coping strategies Significant negative correlation between blood Phe and maternal mealtime mood at 15 and 18 m **Maternal coping strategies—**significantly higher use in mothers of children with PKU vs controls at child ages 15 m and 24 m Low level of anxiety scores in both groups of mothers (no significant difference between two groups) Mothers of children with PKU showed more variable anxiety (larger SD)
Haitjema et al., 2022 [38]	Caregivers of children with PKU on diet (*n* = 43) vs. Caregivers of Healthy children (*n* = 50)	Parental mealtime behaviour	**PKU-specific questionnaire:** 18 questions; assesses social restrictions and eating problems not covered by the BPFAS (e.g., going out for dinner, child’s first sleepover, school trips;) 5-point Likert scale (“never” 1 to “always” 5 and “not stressful” 1 to “very stressful” 5); parent-reported **Behavioural Paediatrics Feeding Assessment Scale (BPFAS; validated) ^b^:** 35 items: 25 items on child behaviour and 10 items on parent feelings around mealtime behaviour; 5-point Likert scale (“never” 1 to “always” 5; parent-reported; higher scores = more problems	Caregivers of children with PKU on diet found it more difficult to offer a varied diet to their child and experienced more stress when eating an evening meal outside the home and during vacation (*p* < 0.05) Caregivers of children with PKU on diet were stricter in their approach to spillage of food during mealtime than controls (*p* < 0.005) A rigid parental approach to dietary treatment was less often correlated with optimal blood Phe control **PKU children on diet alone vs. healthy controls:** Total BPFAS parent behaviour/feeling score (median, range): 18 [10–34] vs. 14 [10–29]; *p* = 0.015 Caregivers of children with PKU on diet were more frustrated, angry and anxious when feeding their child compared with controls (*p* < 0.05) Caregivers of children on diet felt less confident that their child received sufficient food compared with controls (*p* < 0.05) Caregivers of children with PKU on diet more often felt that their child’s eating pattern had a negative influence on their general health compared with controls (*p* < 0.05) Caregivers of children with PKU on diet more often prepared alternative food if their child did not like what was being served compared with controls (*p* < 0.05) **PKU total sample:** Caregivers of younger children (1–7 y) with PKU reported getting more frustrated during mealtime and more often coaxed their child to get the child to take a bite than caregivers of older children (8–16 y) (*p* < 0.05)
Rice et al., 2023 [46]	Children with PKU (*n* = 39)	Parental anxiety/stress	**Survey questionnaire (non-validated), medical notes**	**52% (*n* = 11/21) of parents reported challenges** during the complementary feeding process, including food refusal, protein calculation, and **anxiety** around maintaining good blood Phe levels **81% (*n* = 17/21) of mothers reported stress** during the complementary feeding period: - Infant’s fussy eating/food refusal - Calculating the protein content in food - Knowing how to count leftover portions - Juggling spoon feeds and PS - Educating family members/other caregivers - Wrong foods given - Preparation of food, always having food with them - Travel/planning ahead - Reflux—food reactions/intolerances - Worrying about blood Phe levels going in/out of range
**C. Sensory and Food-Related Perceptions:** Outcomes related to taste/food preferences				
Bentovim et al., 1970 [41]	Children with PKU (*n* = 12)	Food preference, fussiness	**Probe questions related to behaviour of the child in relation to diet 7)**: 5-point Likert scale; parent-reported; to assess: appetite, greediness, finickiness, acceptance of the dietary restrictions	Unusual appetite for savoury foods in most (e.g., salad cream on special spaghetti, raw mushrooms, pickled onions, and curry sauce) Half the children, particularly the older ones, disliked so many vegetables in the diet; some persuaded other children to give them forbidden sweets and stole food Younger children were remarkably more accepting of the restrictions and appeared uninterested in other foods, asking permission to accept sweets
MacDonald et al., 1994 [34]; MacDonald et al., 1997 [31]	Children with PKU (*n* = 15) vs. Healthy children (*n* = 15)	Food preferences	**3-day food diary**	**Dislike for sweet foods** (e.g., sweets/chocolates, cakes/biscuits, puddings): ***s***ignificantly more common in PKU vs. control **Favourite foods of PKU group:** crisps, pickled onions and olives
Evans et al., 2016 [35]; Evans et al., 2018 [32]	Children with PKU and their parents (*n* = 35) vs. Healthy children and their parents (*n* = 35)	Taste/food preferences	**Food rating:** Children tasted *n* = 10 blinded puréed foods in random order; 7-point hedonic scale (“super yummy” to “super yucky”) **Food ranking:** Ordering of food from “most” to “least” liked	**Taste preference ratings:** Children in both PKU and control groups preferred sweet items to savoury, bitter, or sour foods **Food group ranking:** Children with PKU ranked vegetables as a group higher than controls (mean ranking: 5.6 vs. 6.3; *p* = 0.05) Control children ranked fruits as a group higher than children with PKU (mean ranking: 3.7 vs. 4.6; *p* < 0.05) No significant difference between PKU vs. control children when foods were grouped as bitter (broccoli, cauliflower, coffee) and sweet (apple, banana, custard) **Overall:** early and ongoing exposure to bitter-tasting L-amino acid supplements did not appear to shape taste perception in PKU

Abbreviations: PKU: phenylketonuria; min: minutes; m: months; y: years; EAT-26: Eating Attitudes Test-26; BPFAS: Behavioural Paediatrics Feeding Assessment Scale; PS: protein substitute; Phe: phenylalanine; CHIP: Coping Health Inventory for Parents; SD: standard deviation; BH4: sapropterin. ^a^ Some questions were eliminated from the **Eating Attitudes Test (EAT-26)** because they would be answered abnormally by individuals with PKU. Additional questions were added to elucidate the cause of behaviour that might otherwise be considered “disordered” in the general population. ^b^ Two questions in the “**Behavioural Paediatrics Feeding Assessment Scale (BPFAS**)”, regarding milk and meat/fish, were removed. ^c^ **Coping Health Inventory for Parents (CHIP) subsets:** Subset 1—Maintaining family integration, cooperation and optimism; Subset 2—Maintaining social support, self-esteem and psychological stability; Subset 3—Understanding the medical situation through communication with other parents/medical staff.

**Table 5:** Data on factors potentially contributing to disordered eating in patients with PKU from included studies.

Reference	Sample (Population vs. Comparator)	Outcomes	Method of Assessment	Key Findings
**A. Early Feeding Skill:** Outcomes related to feeding development				
MacDonald et al., 1994 [34] MacDonald et al., 1997 [31]	Children with PKU (*n* = 15) vs. Healthy children (*n* = 15)	Feeding skills	**Mealtime positive-negative rating scale:** 5-point Likert scale	**Feeding skills: drinking from infant bottle**: significantly higher in PKU vs control (67% vs. 7%)
Evans et al., 2017 [44]	Infants with PKU (*n* = 31)	Feeding skills	**Patient medical records from weaning age until 2 y**	**Age at introduction of solid foods:** median 17 weeks **Delayed introduction of more textured foods:** 32% **(**on >1 occasion in 10%); median 9 months [range: 7–20 months]; significantly higher in first-born infants [80% vs. 38%], infants with food refusal during teething [80% vs. 29%], and having no older siblings [0% vs. 43%])
Evans et al., 2019a [36]; Evans et al., 2019b [33]	Infants with PKU (*n* = 20) vs. Healthy controls (*n* = 20)	Feeding skills	**Feeding Development Diary ^a^** (non-validated, qualitative): 3-day diary; parent-reported; Assessments: monthly to 12 m, then at 15, 18 and 24 m of age **Feed-time (non-validated):** 9 questions, 7-point Likert scale; parent-reported; Assessments: at weaning, 8, 12, 15, 18 & 24 m of age	**Age at weaning:** significantly earlier in PKU vs. control (4.1 vs. 5 m) **Self-finger feeding:** significantly later in PKU vs. controls (6.5 vs. 5.7 m) **Prolonged bottle-feeding (Phe-free PS) and parental spoon feeding:** significantly higher in PKU vs. controls
Rocha et al., 2023 [47]	Children with PKU (*n* = 40) vs. Healthy children (*n* = 46)	Feeding skills	**Questionnaire (non-validated), clinical records**	**Feeding autonomy:** significantly lower in infants with PKU vs. controls (70 vs. 94%; *p* = 0.005) **Use of baby bottle** significantly more common in infants with PKU vs. controls (100% vs 87%)
**B. Social Impact:** Outcomes related to the social dimensions of eating and disease				
MacDonald et al., 1994 [34]; MacDonald et al., 1997 [31]	Children with PKU (*n* = 15) vs. Healthy children (*n* = 15)	Mealtime environment	**Mealtime positive-negative rating scale:** 5-point Likert scale **Video of a midday meal**	Children in the PKU group were more likely to be given their midday meal separate from the rest of the family (PKU group 67%, control 20%; *p* < 0.05) **Eating separately from family:** significantly higher in PKU vs. control (56% vs. 7%)
Roberts et al., 2017 [43]	Adults with PKU on diet (*n* = 8)	Social eating experiences	**Semi-structured interviews on impact of PKU throughout life (non-validated):** 30–90 min sessions	**Childhood eating experience:** Many reported feeling different from peers in social eating situations (e.g., school, parties), often linked to perceiving PKU as a burden Some described feelings of isolation and distress, particularly when questioned about their restricted diet **Adolescent eating experience:** A period of reduced dietary compliance and increased difficulties managing the condition Some reported using dietary rules as an outlet for frustration **Adult eating experience:** Some reported that eating in social contexts remained challenging (e.g., lack of suitable meals), leading to avoidance of dining out Others reported good adaptation and did not perceive social eating as problematic **Positive parental support was linked to better adult outcomes**, including **fewer difficulties with social eating** (e.g., inclusion at family meals and facilitation of skill development) Early experiences of social exclusion or limited support were associated with poorer psychological wellbeing and dietary non-adherence in adulthood
Evans et al., 2019a [36]; Evans et al., 2019b [33]	Infants with PKU (*n* = 20) vs. Healthy controls (*n* = 20)	Mealtime environment	**Feeding development diary ^a^** (non-validated, qualitative): 3-day diary; parent-reported; Assessments: monthly to 12 m, then at 15, 18 and 24 m of age	**Feeding environment:** after 15 m of age, children with PKU were more likely to be fed separately from the rest of the family
Haitjema et al., 2022 [38]	Caregivers of: children with PKU on diet (*n* = 43) vs. healthy children (*n* = 50)	Mealtime environment	**PKU-specific questionnaire ^b^:** 18 questions; assesses social restrictions and eating problems; 5-point Likert scale (“never” 1 to “always” 5 and “not stressful” 1 to “very stressful” 5); parent-reported	**PKU children on diet alone vs. healthy controls** Children with PKU on diet were more likely to eat their evening meal separate from the rest of the family compared with controls (*p* < 0.05)
**C. Physical Symptoms and Medical Comorbidities:** Outcomes related to eating and gastrointestinal symptoms				
MacDonald et al., 1994 [34]; MacDonald et al., 1997 [31]	Children with PKU (*n* = 15) vs. Healthy children (*n* = 15)	Gastrointestinal symptoms	**Mealtime positive-negative rating scale:** 5-point Likert scale	**Gastrointestinal symptoms:** significantly more frequent in PKU vs. control (60% vs. 7%; *p* < 0.05); vomiting 7% vs. 0%; constipation 27% vs. 0%; diarrhoea 27% vs. 0%; abdominal pain and colic 20% vs. 7%.
Bilder et al., 2017 [37]	Adults with PKU (*n* = 2242) vs. Adults with Diabetes (Type I/II; *n* = 4334) vs. General population (*n* = 11.981);	Physical/neurological disorders potentially related to eating disorders/disordered eating	**ICD-9 codes on database claims:** Prevalence ratio (PR)	**PR of disorders in PKU vs. Diabetes vs. Healthy controls:** Sleep disturbances: 9.0% vs. 10.8% vs. 4.9% Movement disorders/tremors: 3.3% vs. 3.7% vs. 1.4% Fatigue and malaise: 1.0% vs. 1.0% vs. 0.5% Migraine and headache: 8.7% vs. 9.2% vs. 5.5% Epilepsy and convulsions: 2.4% vs. 4.7% vs. 1.5% Pain disorders: 57.4% vs. 59.6% vs. 38.4% Multiple sclerosis: 0.3% vs. 0.5% vs. 0.2% Dementia: 0.6% vs. 0.8% vs. 0.3% **PR for other disorders: PKU vs. Diabetes:** No significant difference between PKU vs. Diabetes **PR for other disorders: PKU vs. general population:** Individuals with PKU had significantly higher PR for all categories
Evans et al., 2017 [44]	Infants with PKU (*n* = 31)	Gastrointestinal symptoms	**Patient medical records from weaning age until 2 y**	6% **self-induced vomiting** with food (3% on >1 occasion)
Evans et al., 2019a [36]; Evans et al., 2019b [33]	Infants with PKU (*n* = 20) vs. Healthy controls (*n* = 20)	Gastrointestinal symptoms	**Feeding Development Diary ^a^** (non-validated, qualitative): 3-day diary; parent-reported; Assessments: monthly to 12 m, then at 15, 18 and 24 m of age	**Gastrointestinal symptoms (flatulence, burping, retching):** more frequent in PKU vs. controls at all ages
Rocha et al., 2023 [47]	Children with PKU (*n* = 40) vs. Healthy children (*n* = 46)	Gastrointestinal symptoms	**Questionnaire (non-validated), clinical records** **EBAI (Brazilian Infant Feeding Scale):** 14 items; 7-point Likert scale; scores 61–65: mild difficulties, 66–70: moderate difficulties, >70: serious difficulties	**Gastrointestinal symptoms:** similar between PKU vs. controls (48% vs. 33%) **EBAI score** was significantly related to gastrointestinal symptoms score in children with PKU (symptoms: 59 vs. no symptoms: 50; *p* = 0.003)
Albers et al., 2026 [52]	Adults with PKU on diet (*n* = 36), on BH4 treatment (*n* = 3) and on pegvaliase treatments (*n* = 2)	Physical/neurological disorders potentially related to eating disorders/disordered eating	**Patient medical records** **PKU-QoL Questionnaire ^c^:** 65 questions, 35 domains, 4 modules; 4–6-point Likert scale; lower scores = lower impact on QoL; ≤25: Little to no impact, 25–50: Moderate impact, 50–75: Substantial impact, ≥75: Very strong impact (very severe and/or very frequent symptoms)	**Neurological disorders (migraines, epilepsy)—**14.6% (*n* = 6/41) **Patients with vs. without psychiatric disorders:** individuals with PKU with psychiatric disorders reported stomach aches more frequently (*p* < 0.05).
**D. Psychological and Psychiatric Outcomes:** Potentially related to eating disorder/disordered eating behaviour				
Bentovim et al., 1970 [41]	Children with PKU *n* = 12	Behavioural difficulties	**Interview**	Common symptoms—tantrums, anxiety, fearfulness, clinginess, night terrors and obsessive behaviours *n* = 6/12 (50%) mild-to-moderate emotional disorder
Antisdel et al., 2000 [39]	Children with PKU (*n* = 30) vs. Children with Type I Diabetes (*n* = 54) vs. Healthy children (n/r) (all females)	**Psychological adjustment to disease**	**Psychological Adjustment to Disease (ATT-19; modified for use in PKU):** 5-point Likert scale (“completely disagree” 1 to “completely agree” 5); higher scores = more positive adjustment	**Psychological adjustment:** significantly poorer in patients with PKU who had symptoms of disordered eating compared with non-symptomatic
Bilder et al., 2017 [37]	Adults with PKU (*n* = 2242) vs. Adults with Diabetes (Type I/II; *n* = 4334) vs. General population (*n* = 11.981);	Psychiatric, psychological, and cognitive disorders potentially related to eating disorders/disordered eating	**ICD-9 codes on database claims:** Prevalence ratio (PR)	**PR of psychiatric disorders in PKU vs. Diabetes vs. Healthy controls:** Anxiety: 13.8% vs. 13.8% vs. 8.8% Depression: 16.3% vs. 19.7% vs. 10.2% OCD: 0.9% vs. 0.6% vs. 0.3% Personality disorders: 1.0% vs. 1.3% vs. 0.7% Substance abuse: 2.1% vs. 3.9% vs. 2.4% Alcohol dependency: 1.2% vs. 2.6% vs. 1.5% Autism: 0.7% vs. 0.2% vs. 0.1% ADD/ADHD: 2.9% vs. 2.2% vs. 1.7% Phobias: 0.7% vs. 0.8% vs. 0.4% Behaviour/conduct disorders: 1.3% vs. 1.2% vs. 0.5% Psychosis/schizophrenia: 1.8% vs. 2.4% vs. 1.1% Bipolar disorder: 3.7% vs. 5.4% vs. 2.9% Tourette/Tic disorders: 0.3% vs. 0.1% vs. 0.0% Intellectual disabilities: 1.9% vs. 0.7% vs. 0.5% Cognitive and/or personality changes secondary to general medical condition: 1.3% vs. 1.4% vs. 0.4% Behaviour/conduct: 1.3% vs. 1.2% vs. 0.5% **PR for psychiatric disorders: PKU vs. Diabetes:** Individuals with PKU had significantly higher PR for autism spectrum disorder, obsessive–compulsive disorder, intellectual disabilities, and ADHD Individuals with PKU had significantly lower PR for depression, substance abuse, alcohol dependency, sleep disturbances, psychosis/schizophrenia, and bipolar disorder No significant difference between PKU vs. Diabetes in PR for anxiety, personality disorders, phobias, behaviour and conduct disorders **PR for psychiatric disorders: PKU vs. general population:** Individuals with PKU had significantly higher PR for nearly all psychiatric categories except personality disorders, substance and alcohol misuse
Roberts et al., 2017 [43]	Adults with PKU on diet (*n* = 8)	**Psychological disorders and psychological wellbeing**	**Patient medical history** **Mini-International Neuropsychiatric Interview (M.I.N.I.) (English Version 6.0.0)** **Semi-structured interviews on impact of PKU throughout life (non-validated):** 30–90 min sessions	*n* = 3/8 (38%) were assessed on the M.I.N.I. as experiencing significant psychological disorders in their lifetime; Major depressive disorder (*n* = 1; 12.5%) Bipolar affective disorder, with a current depressive episode (*n* = 1; 12.5%) **Non-adherence, metabolic control and psychological effects:** Women described an association between being off-diet or having elevated Phe levels and negative psychological and physical effects, with this relationship operating bidirectionally **Overall**: these factors appear to interact over time, contributing to a cumulative long-term impact on wellbeing
Pinto et al., 2025 [49]	Adolescents with PKU on diet only (*n* = 30) and on diet + BH4 treatment (*n* = 3)	**Anxiety and depression**	**Hospital Anxiety and Depression Scale (HADS; validated):** 2 subscales: HADS-A (anxiety) and HADS-D (depression); each scored from 0 to 21; Normal: 0–7, Borderline abnormal: 8–10, Abnormal: 11–21 **Euroqol-5D-5L (EQ-5D-5L) Scale:** Self-completed; 5 dimensions rated on a 5-point Likert scale (mobility, self-care, usual activities, pain/discomfort and anxiety/depression); with a maximum general health status classification score of 100 perceived by patients **Medical notes**	**Anxiety and depression:** mean anxiety scores were nearly twice as high in female participants compared to males 40% (10/25) abnormal, 8% (2/25) borderline anxiety scores 12% (3/25) abnormal, 12% (3/25) borderline depression scores No significant correlation between anxiety, depression, and mean blood Phe (anxiety: r = 0.1942 and depression: r = 0.3713; *p* > 0.05) Higher blood Phe levels appeared to correlate with elevated scores on several HADS items (*p* > 0.05) **Feelings of anxiety and depression:** 20% (5/25) had moderate anxiety and depression; 16% (4/25) had severe, and 4% (1/25) had extreme feelings of anxiety and depression 6% (2/33) attended a special school due to autism as a co-morbidity 12% (4/33) had autism and/or attention deficit hyperactivity disorder (ADHD) 21% (7/33) had depression and anxiety 15% (5/33) had low mood with suicidal or self-harm thoughts School anxiety (*n* = 1) and behaviour issues (*n* = 1) were documented Methylphenidate for ADHD treatment (*n* = 1) and anti-depressants (*n* = 2) were prescribed
Albers et al., 2026 [52]	Adults with PKU on diet (*n* = 36), on BH4 treatment (*n* = 3) and on pegvaliase treatments (*n* = 2)	**Psychiatric disorders and psychological well-being**	**Patient medical records** **WHO-5 Wellbeing Index (WHO-5):** 5 questions scored between 0 and 5 (total score 0–25); to assess general well-being and mental health; completed annually; lower scores = poorer well-being; score < 13 = a potential risk of depression **PKU-QoL Questionnaire ^c^:** 65 questions, 35 domains, 4 modules; 4–6-point Likert scale; lower scores = lower impact on QoL; ≤25: Little to no impact, 25–50: Moderate impact, 50–75: Substantial impact, ≥75: Very strong impact (very severe and/or very frequent symptoms)	**At least one psychiatric diagnosis:** 26.8% (*n* = 11/41) ^d^ **Multiple psychiatric diagnoses:** 14.6% (*n* = 6/41) ^d^: 9.8% (*n* = 4/41) anxiety disorders 7.3% (*n* = 3/41) PTSD 2.4% (*n* = 1/41) hypochondriacal disorders 12.2% (*n* = 5/41) depression 4.9% (*n* = 2/41) drug addiction 7.3% (*n* = 3/41) borderline personality disorder 2.4% (*n* = 1/41) paranoid schizophrenia 2.4% (*n* = 1/41) ADHD **Patients with vs. without psychiatric disorders ^d^:** Higher mean blood Phe 1093 ± 415.3 vs. 774.5 ± 389.7 (*p* = 0.029) Lower Phe within target: 42.9% ± 36.3 vs. 45.0% ± 36.4 (*p* = 0.872) Lower adherence to a calculated diet: 18.2% vs. 50% (*p* = 0.068) More often reported inconsistent or no AAM use (*p* > 0.05) **WHO-5 scores (Mean ± SD):** 16.5 ± 4.8 (range 2–25) (“not depressed”) At transition—16.0 ± 4.0 (range: 7–24) 1st annual FU—16.5 ± 5.6 (range: 2–25) 2nd annual FU—16.2 ± 5.4 (range: 3–21) 3rd annual FU—16.7 ± 5.1 (range: 7–24) **Dietary habits:** “Food temptation, practical impact of protein restriction and feelings of guilt if diet not followed” had the greatest impact on QoL (median scores 25–50 = “moderate”) All other aspects of dietary protein restriction had little or no impact (score < 25) **Patients with vs. without psychiatric disorders:** Individuals with PKU with psychiatric disorders experienced sadness more frequently, whereas slow thinking, aggressiveness, and moodiness were reported less often (*p* < 0.05). For all other PKU-QoL domains, no significant difference was observed
Giret et al., 2026 [53]	*n* = 187 adults with PKU: on diet (*n* = 104), off diet (*n* = 83), on BH4 treatment (*n* = 5) ^e^	**Psychiatric disorders**	**psychiatric history** (clinical diagnosis, specialist requirement, admissions into psychiatric wards, specific therapy) **medical interview** All clinical diagnoses were coded according to ICD10	25.7% (*n* = 48/187) ^f^ had history of psychiatric conditions; 27% (*n* = 41/152) cPKU, 19.2% (*n* = 5) mPKU, 22.2% (*n* = 2/9) mHPA); 21% (*n* = 22/104) on diet, 31.3% (*n* = 26/83) off diet No significant difference in incidence between PKU severities or “on diet vs “off diet” 15.5% (*n* = 29/187) ^f^ depressive episodes; 16.5% (*n* = 25/152) cPKU, 7.7% (*n* = 2/26) mPKU, 22.2% (*n* = 2/9) mHPA 13.4% (*n* = 25/187) ^f^ anxious disorders; 14.5% (*n* = 22/152) cPKU, 11.5% (*n* = 3/26) mPKU 1% (*n* = 2) ^f^ for each of: delirious access, sleep disorders 0.5% (*n* = 1) ^f^ for each of: aggressiveness, claustrophobia, irritability/anger, somnambulism, sadness, OCD, drug suicide attempt

Abbreviations: PKU: phenylketonuria; min: minutes; m: months; y: years; BPFAS: Behavioural Paediatrics Feeding Assessment Scale; PS: protein substitute; Phe: phenylalanine; SD: standard deviation; BH4: sapropterin; EBAI: Brazilian Infant Feeding Scale; PR: prevalence ratio; OCD: obsessive–compulsive disorder; ADHD: Attention Deficit Hyperactivity Disorder; ADD: Attention Deficit Disorder; PTSD: Post Traumatic Stress Disorder; AAM: Amino Acid Mixtures; HADS: Hospital Anxiety and Depression Scale; HADS-A: Hospital Anxiety and Depression Scale (Anxiety); HADS-D: Hospital Anxiety and Depression Scale (Depression); ATT-19: Diabetes Attitude Questionnaire; EQ-5D-5L: EuroQol 5 Dimensions, 5 Levels; WHO-5: World Health Organisation-Five Wellbeing Index; QoL: quality of life; cPKU: classic phenylketonuria; mPKU: mild phenylketonuria; mHPA: mild hyperphenylalaninemia; ICD10: world Health Organisation’s International classification of diseases, 10th revision. ^a^ **Feeding Development Diary**: to assess stage of texture progression, feeding skills, feeding environment, gastrointestinal symptoms, and behavioural difficulties administering weaning PS. **^b^ PKU specific questionnaire**: assesses social restrictions and eating problems not covered by the BPFAS (e.g., going out for dinner, child’s first sleepover, school trips). **^c^ PKU QoL questionnaire**: to assess PKU symptoms—PKU in general, administration of Phe-free PS, dietary protein restrictions. ^d^ Calculations based on early diagnosed patients only, additional data provided by authors. ^e^ Not clear if patients on BH4 were on diet also. ^f^ Unclear if these included patients “on” or “off diet” or on BH4 treatment; however, the authors report that an analysis of the prevalence of psychiatric disorders in patients “on diet” and “off diet” did not find any statistically significant difference (*p* = 0.11).

**Table 6:** Data on disordered eating traits, attitudes, behaviours and other factors potentially associated with or contributing to disordered eating in patients with PKU on pharmacological treatment from included studies.

Reference	Sample (Population vs. Comparator)	Outcomes	Method of Assessment	Key Findings
***A.* Disordered eating assessment tools:** Outcomes related to risk of eating disorders				
Viau et al., 2023 [48]	Adults with PKU on pegvaliase treatment (*n* = 11)	Disordered eating (eating attitudes and behaviours)	**Three Factor Eating Questionnaire Revised-18 (TFEQ-R18) ^a^:** Scores range between 18 and 72; Higher scores = More of the behaviour	**TFEQ-R18 Subscales ^a^**: responders (*n* = 6) vs. partial-responders (*n* = 5): **Uncontrolled eating scores:** - Baseline: 22.0 ± 5.0 vs. 18.0 ± 7.6 - 9 m: 18.2 ± 4.6 vs. 17.0 ± 5.3 - 15 m: 15.6 ± 5.2 vs. 17.6 ± 5.3 **Cognitive restraint scores:** - Baseline: 15.0 ± 2.3 vs. 10.2 ± 0.4 - 9 m: 15.2 ± 4.0 vs. 11.0 ± 4.3 - 15 m: 14.4 ± 3.8 vs. 13.0 ± 4.3 **Emotional eating scores:** - Baseline: 7.0 ± 3.5 vs. 7.0 ± 3.2 - 9 m: 6.8 ± 2.4 vs. 7.6 ± 2.4 - 15 m: 6.2 ± 3.7 vs. 7.0 ± 1.6 **Overall;** **Uncontrolled eating—**Decreased only in responders (by −5 ± 3.6; range: −9 to–2; minimal change in partial responders) **Emotional eating—**Decreased only in responders (by −0.8 ± 1.6; range: −3 to 1; minimal change in partial responders)
			**Emotional Eater Questionnaire (EEQ):** Scores range between 0 and 30; Higher scores = More emotional eating behaviours	**EEQ Scores:** responders (*n* = 6) vs. partial-responders (*n* = 5): Baseline: 11.3 ± 7.4 vs. 15.4 ± 6.5 9 m: 8.6 ± 5.3 vs. 16.0 ± 6.2 15 m: 6.4 ± 4.5 vs. 13.0 ± 5.0 **Overall:** Emotional eating was decreased only in responders (by −5.2 ± 7.2; range: −16 to 4; minimal change in partial responders)
***B.* Limited variety/Selective eating:** Outcomes related to dietary variety and meal frequency				
Thiele et al., 2015 [42]	Children with PKU (*n* = 8); Pre-BH4 (diet only) vs. Post-BH4 (*n* = 5; fully liberalised diet; *n* = 3 partially liberalised diet) vs. Healthy population	Food intake and dietary variety	**3-day diet records**	**Diet only:** individuals allocated a substantial proportion of their total food intake to fruit, vegetables and SLPFs (e.g., low protein bread, pasta and milk substitutes) **BH4 treatment:** individuals changed their eating habits, which remained relatively stable over the long-term follow-up. BH4 treatment was associated with fewer SLPFs and more protein-rich foods (bread, potatoes, pasta, rice, dairy products and meat). **After 2 y of BH4 treatment:** consumption of fruits, vegetables, breads and cereals, meat, fats and sweets were similar to healthy peers; milk and dairy was markedly lower; BH4 patients consumed twice as much potato, pasta and rice compared to their peers; and lower intake of fish
Haitjema et al., 2022 [38]	Caregivers of children with PKU on diet (*n* = 43) vs. Caregivers of children with PKU on BH4 (*n* = 14) vs. Caregivers of Healthy children (*n* = 50)	Food intake and dietary variety	**Behavioural Paediatrics Feeding Assessment Scale (validated) ^b^:** 35 items: 25 items on child’s behaviour and 10 items on parent’s feelings around mealtime behaviour; 5-point Likert scale (“never” [1] to “always” [5]; parent-reported; Higher scores = more problems	BPFAS child behaviour item score: **PKU children on BH4 treatment vs. healthy controls:** No significant difference in terms of vegetable intake frequency between groups (*p* < 0.05) **PKU total sample (diet + BH4 treatment):** Caregivers of younger children (1–7 y) indicated that their child ate more vegetables than older children (8–16 y) (*p* < 0.05)
Yilmaz Nas et al., 2026 [51]	Children with PKU non-responsive to BH4 (diet-only treated; *n* = 12) vs. Children with PKU responsive to BH4 (diet + BH4 treated; *n* = 21)	Food intake and dietary variety	**Food frequency questionnaire (FFQ; validated for PKU):** 89-item **Dietary Burden of Care Questionnaire (non-validated):** 16-items; open-ended questions	**BH4 non-responsive group**: showed no significant within-group changes in intake of any food category over 24 months. **BH4 responsive group:** in contrast, was strongly associated with changes in dietary patterns including a marked shift from SLPFs toward higher-protein foods over 24 months. Responders significantly increased consumption of cow’s milk, regular cheese, meat/fish/eggs, regular bread, and regular pasta, whilst most SLPFs declined substantially over the same period. Intake of regular foods varied according to metabolic capacity, with individuals tolerating >40 g/day Phe consuming the highest amounts. Consumption of chips/processed potato, vegetables and fruit remained stable, indicating that changes were specific to protein-containing food categories rather than general dietary behaviour. Two-thirds (14/21; 67%) of responders also reported reduced or minimal use of SLPFs **Non-responsive participants:** showed no improvement in dietary flexibility or spontaneity, with all individuals (12/12; 100%) maintaining a rigid dietary pattern and a continued heavy daily dependence on SLPFs. **BH4-responsive participants:** demonstrated marked behavioural change: all responders (21/21; 100%) reported greater dietary flexibility and spontaneity, characterised by increased food variety and freedom and reduced use of SLPF.
***C.* Food and general neophobia:** Outcomes related to reluctance to eat new or different foods				
Viau et al., 2021 [45]	Adults with PKU on pegvaliase treatment (*n* = 18)	Food neophobia	**Modified Food Neophobia Scale (FNS) (adapted for PKU):** 9-item; self-reported; Total score: High: 9–27 Moderate: 28–45 Low: 46–63	**FNS scores:** **“Low” level neophobia—**47% **“Moderate level neophobia—**35% **“High” level neophobia—**18% **Overall: Moderate to high food neophobia**—In 53% of participants on pegvaliase treatment, it enabled a normal dietary protein intake without restrictions No correlation between FNS scores and duration of pegvaliase treatment or overall diet quality as measured by the Healthy Eating Index (HEI)
Viau et al., 2023 [48]	Adults with PKU on pegvaliase treatment (*n* = 11)	Food neophobia	**Modified Food Neophobia Scale (FNS; adapted for PKU):** 9-item; High: Score 9–27, moderate: score 28–45, and low: score 46–63	**FNS Scores:** responders (*n* = 6) vs. partial-responders (*n* = 5): Baseline: 40.2 ± 11.1 vs. 45.6 ± 8.3 9 m: 52.0 ± 6.0 vs. 39.8 ± 14.2 15 m: 48.8 ± 8.0 vs. 42.0 ± 12.3 **Food neophobia—**Decreased only in responders (FNS score increased by 8.6 ± 6.7; range: 2 to 18; minimal change in partial responders)
Yilmaz Nas et al., 2026 [51]	Children with PKU non-responsive to BH4 (diet-only treated; *n* = 12) vs. Children with PKU responsive to BH4 (diet + BH4 treated; *n* = 21)	Food and general neophobia	**Food Neophobia Scale (adapted from adult FNS):** 9 items for food neophobia and 5 items for general neophobia; 7-point scale (“always” 1 to “never” 7)	**Food neophobia score (baseline vs. 24 m):** BH4 non-responsive: 34.2 ± 14.5 vs. 37.6 ± 11.3 (*p* = 0.295) BH4 responsive: 28.5 ± 10.3 vs. 36.3 ± 11.7 (*p* = 0.005) **General neophobia score (baseline vs. 24 m):** BH4 non-responsive: 16.9 ± 8.8 vs. 18.1 ± 8.5 (*p* = 0.531) BH4 responsive: 14.5 ± 8.6 vs. 18.6 ± 9.1 (*p* = 0.091) **Overall:** BH4 responsive group was less food neophobic
***D.* Mealtime Behaviour:** Outcomes related to eating behaviour and emotions				
Viau et al., 2021 [45]	PKU adults on pegvaliase treatment (*n* = 18)	Enjoyment and aesthetic appreciation for food	**Epicurean eating tendencies (EET) scale:** Total score ranges between 7 and 49; self-reported; Higher scores = more prominent Epicurean eating tendencies	**Aesthetic appreciation of food:** significantly associated with lower FNS scores; indicating those less fearful of trying new foods experienced increased enjoyment of food
Haitjema et al., 2022 [38]	Caregivers of children with PKU on diet (*n* = 43) vs. Caregivers of children with PKU on BH4 (*n* = 14) vs. Caregivers of Healthy children (*n* = 50)	Enjoyment of food	**Behavioural Paediatrics Feeding Assessment Scale (BPFAS; validated) ^b^:** 35 items: 25 items on child’s behaviour and 10 items on parent’s feelings around mealtime behaviour; 5-point Likert scale (“never” [1] to “always” [5]; parent-reported	**PKU total sample (diet + BH4 treatment):** Caregivers of younger children (1–7 y) reported that their children enjoyed food less than caregivers of older children (8–16 y) (*p* < 0.05)
Viau et al., 2023 [48]	Adults with PKU on pegvaliase treatment (*n* = 11)	Enjoyment and aesthetic appreciation for food	**Epicurean Eating Tendencies (EET) Scale:** Scores range between 7 and 49; Higher scores = more prominent Epicurean eating tendencies	**EET Score:** responders (*n* = 6) vs. partial-responders (*n* = 5): - Baseline: 27.0 ± 6.1 vs. 33.0 ± 5.8 - 9 m: 35.2 ± 5.5 vs. 30.4 ± 9.1 - 15 m: 35.8 ± 7.3 vs. 33.4 ± 8.2 **Enjoyment and appreciation for food:** increased only in responders (by 8.8 ± 11.1; range: −4 to 26; minimal change in partial responders)
***E.* Caregiver Factors:** Emotional and Psychological Aspects; Practices and Coping Strategies at Mealtimes				
Haitjema et al., 2022 [38]	Caregivers of: Children with PKU on BH4 (*n* = 14) vs. Healthy children (*n* = 50)		**Behavioural Paediatrics Feeding Assessment Scale (BPFAS; validated) ^b^:** 35 items: 25 items on child’s behaviour and 10 items on parent’s feelings around mealtime behaviour; 5-point Likert scale (“never” 1 to “always” 5; parent-reported; Higher scores = more problems	**PKU children on BH4 treatment vs. healthy controls:** Total BPFAS child behaviour (median, range): 45 [31–70] vs. 14 [10–29]; *p* > 0.05 **PKU children on BH4 treatment vs. healthy controls:** Total BPFAS parent behaviour/feeling score (median, range): 17.5 [10–25] vs. 14 [10–29]; *p* > 0.05 Caregivers in the BH4 group more often had the feeling that their child’s eating pattern might have a negative influence on the child’s general health than controls (*p* < 0.05)
Yilmaz Nas et al., 2026 [51]	Children with PKU non-responsive to BH4 (diet-only treated; *n* = 12) vs. Children with PKU responsive to BH4 (diet + BH4 treated; *n* = 21)		**Hospital Anxiety & Depression Scale (HADS) (validated):** 14-item psychological wellbeing tool; 7-items each for anxiety and depression; 4-point Likert scale (0–3); Normal: 0–7, Borderline: 8–10, Abnormal: 11–21	**HADS anxiety score of caregivers (median [IQR]; baseline vs. 24 m):** BH4 non-responsive: 8 [6–10] vs. 7 [5–8] (*p* = 0.784) BH4 responsive: 4 [2–7] vs. 2 [0–4] (*p* = 0.104) **HADS depression score of caregivers (median [IQR]; baseline vs. 24 m):** BH4 non-responsive: 8 [5–14] vs. 7 [3–12] (*p* = 0.232) BH4 responsive: 4 [2–7] vs. 1 [0–3] (*p* = 0.013) **Overall:** depression scores of caregivers of children with BH4 responsive children were significantly improved after BH4; No difference between BH4 responsive vs. non-responsive groups in terms of anxiety and depression scores.
***F.* Social Impact:** Outcomes related to the social dimensions of eating and disease				
Viau et al., 2023 [48]	Adults with PKU on pegvaliase treatment (*n* = 11)	Social eating restrictions	**Epicurean Eating Tendencies (EET) Scale:** Scores range between 7 and 49; Evaluates aesthetic appreciation for food (e.g., sensory aspects and the symbolic value of food); Higher scores = more prominent Epicurean eating tendencies	**Epicurean eating tendencies score:** increased by 8.8 ± 11.1; range: −4 to 26 for pegvaliase responders **Social aspects of eating improved on pegvaliase** associated with removal of dietary protein restrictions
Yilmaz Nas et al., 2026 [51]	Children with PKU non-responsive to BH4 (diet-only treated; *n* = 12) vs. Children with PKU responsive to BH4 (diet + BH4 treated; *n* = 21)	Impact on family of chronic disease; social eating restrictions	**Dietary Burden of Care Questionnaire (non-validated):** 16-items; open-ended questions	BH4 non-responsive: **School food provision:** packed lunches rather than school meals in 8/12 (67%) **Eating out:** infrequent in 3/12 (25%) **Social participation and sleepovers:** occasionally, lots of planning required (8/12; 67%); sleepovers allowed in 33% (4/12) BH4 responsive: **School food provision:** packed lunches in 13/18 (72%); school meals in 5/18 (28%) **Eating out:** frequent (weekly/monthly) in 21/21 (100%); conflicts rare (3/21; 14%) **Social participation and sleepovers:** attended with minimal restrictions in 18/21 (86%); sleepovers allowed in 13/21 (62%)
***G.* Psychological and Psychiatric Outcomes:** Potentially related to eating disorder/disordered eating behaviour				
Yilmaz Nas et al., 2026 [51]	Children with PKU non-responsive to BH4 (diet-only treated; *n* = 12) vs. Children with PKU responsive to BH4 (diet + BH4 treated; *n* = 21)	Anxiety and depression	**Hospital Anxiety & Depression Scale (HADS) (validated):** 14-item psychological wellbeing tool; 7-items each for anxiety and depression; 4-point Likert scale (0–3); Normal: 0–7, Borderline: 8–10, Abnormal: 11–21	**Overall:** anxiety scores of BH4-responsive adolescents were significantly increased over time after 2 y on BH4; No difference between BH4-responsive vs. non-responsive groups in terms of anxiety and depression scores.

Abbreviations: PKU: phenylketonuria; m: months; y: years; NS: not statistically significant; IQR: interquartile range; BH4: sapropterin; TFEQ-R18FFQ: Three Factor Eating Questionnaire Revised; EEQ: Emotional Eater Questionnaire; BPFAS: Behavioural Paediatrics Feeding Assessment Scale; EET: Epicurean Eating Tendencies; SLPF: special low protein foods; FFQ: food frequency questionnaire; FNS: Food neophobia scale; Phe: phenylalanine; HADS: Hospital Anxiety and Depression Scale. ^a^ **Three Factor Eating Questionnaire Revised-18 (TFEQ-R18) subscales:** (1) Cognitive restraint; (2) Uncontrolled eating; and (3) Emotional eating. One question in the uncontrolled eating domain was changed for PKU. ^b^ Two questions in the “**Behavioural Paediatrics Feeding Assessment Scale (BPFAS)**”, regarding milk and meat/fish, were removed.

**Table 7:** Summary of possible contributing or protective factors for disordered eating and eating/feeding difficulties in patients with PKU.

References	Contributing (Negative) Factors	Protective (Positive) Factors
Bentovim et al., 1970 [41]	Unpalatability of PS and SLPFs (taste/smell/texture) Forcing, smacking when administering PS to children Unusual appetite Maternal anxiety and tension around mealtimes Presence of emotional disorders (e.g., temper tantrums, anxiety, fearfulness, dependent clinging, night terrors)	Support from metabolic clinic
MacDonald et al., 1994 [34]; MacDonald et al., 1997 [31]	Possible altered taste preferences due to bitter taste of L-amino acid PS Coercive mealtime management: parental pressure to eat certain foods Mismatch between parental expectations and child’s physiological cues: exacerbating feeding difficulties and contributing to mealtime conflict Caregiver overestimation of the volume of food children should consume: leading to prompting, coaxing and persistent offering of food when the child is not hungry Lack of variety and unpalatability of foods offered Lack of praise or pleasant conversation about food Eating in isolation Satiety value of PS (effect on appetite) Gastrointestinal symptoms (e.g., constipation, abdominal pain)	Repeated social reinforcement and exposure to new foods Reduced energy content of PS Changing the administration method of PS to reduce gastrointestinal symptoms, e.g., small doses at regular intervals Practical advice and support for parents in overcoming common feeding difficulties Involvement of a psychologist or home visiting specialist metabolic nurse working directly with a dietitian
Antisdel et al., 2000 [39]	Coping with and adhering to the highly restrictive prescribed diet Necessity to consume protein substitutes Internal conflict between striving for health (e.g., maintaining metabolic control, adhering to dietary recommendations) and pursuing thinness or attractiveness	Regular focus on health for adolescent and young women Disorder-specific camps that may improve self-esteem
Thiele et al., 2015 [42]	Timing of BH4-responsiveness testing and age at treatment initiation: later introduction of sapropterin therapy may increase the likelihood of entrenched food aversions, particularly towards protein-rich foods, e.g., fish and eggs	Initiating treatment with sapropterin at a younger age: supports more flexible sensory learning and smoother incorporation of higher-protein foods before long-standing avoidance patterns become established
Evans et al., 2016 [35]; Evans et al., 2018 [32]	Food neophobia	Higher maternal levels of education: associated with a broader range of foods, reflecting increased nutrition knowledge and confidence in feeding practices
Bilder et al., 2017 [37]	Presence of neuropsychiatric comorbidities: OCD, autism, intellectual disabilities, Tourette syndrome, behavioural or conduct disorders, cognitive or personality changes, and dementia; may affect sensory processing, rigidity around routines, impulse control, emotional regulation, and capacity to adapt to dietary demands	n/r
Roberts et al., 2017 [43]	Presence of psychological disorders (e.g., major depressive disorder, bipolar affective disorder) Social isolation: early and repeated experiences of social exclusion, feeling different from peers in social eating situations, leading to feelings of isolation, self-consciousness and distress, especially when questioned about their diet Negative attitudes towards PKU and its diet: viewing PKU as a burden Dietary non-compliance in adulthood: being off diet or having high blood Phe levels was associated with negative psychological and physical effects	Parental support: providing PKU-friendly foods that resemble those eaten by peers, including children in family meals, and actively facilitating the development of food-related skills. Positive attitudes towards the PKU diet: internalising constructive, balanced views of dietary treatment, seeing it as manageable, purposeful, or beneficial, improves engagement with dietary requirements Provision of PKU knowledge to school peers: can reduce stigma, enhance understanding, and foster a more inclusive social environment, mitigating feelings of isolation
Evans et al., 2017 [44]	Age of weaning: later weaning (>4.2 m of age) is associated with a higher incidence of feeding difficulties Illnesses during the weaning period, e.g., vomiting, diarrhoea, upper-respiratory infections, ear infections, are frequently associated with temporary PS or food refusal, disrupting feeding routines and slowing progress with new foods Previous experience of managing a PKU infant: first-born children or those without older siblings with PKU tend to experience more feeding difficulties during weaning. Repeated offering of familiar foods rather than new foods: limits exposure to new tastes and textures, reducing dietary variety Poor caregiver technique or inconsistent routines for administering weaning PS: inappropriate timing (e.g., immediately after meals when the child is full or when tired), and inconsistent daily schedules	Age of weaning: earlier weaning (<4.2 m of age) is associated with fewer feeding difficulties * Timing of PS in relation to food intake: may improve intake by avoiding competition with satiety from solid foods.
Evans et al., 2019a [36]; Evans et al., 2019b [33]	Age of weaning: delaying beyond 10 m of age is associated with a higher risk of food refusal, fussy/selective eating, delayed development of self-feeding skills, poorly established mealtime routines, and reduced dietary variety; prolonged reliance on milk-based feeding may limit exposure to new tastes and textures Feeding environment: eating separately from the rest of the family can reduce modelling of positive eating behaviours and limit exposure to a wider range of foods Presence of gastrointestinal symptoms: symptoms, e.g., flatulence, burping, retching, abdominal discomfort, or bloating can negatively affect appetite and willingness to eat Maternal anxiety and stress: can influence feeding interactions, leading to increased pressure, inconsistent responses to refusal, or heightened tension during meals.	Periodic psychological reviews of parental stress, anxiety, and coping strategies: enabling timely support Age of weaning: around 4–5 m of age is associated with better acceptance and fewer early feeding difficulties
Luu et al., 2020 [40]	Poor metabolic control/poor dietary management: increased food preoccupation, binge-eating behaviours, perceived pressure to consume more food or PS, greater weight-related concerns and weight-control behaviours Dietary protein restrictions: increased food preoccupation	n/r
Viau et al., 2021 [45]	Food neophobia: reluctance to introduce high-protein foods post pegvaliase therapy	Assessment of food neophobia, sensory sensitivities and behavioural barriers to introducing high-protein foods during diet liberalisation after pegvaliase treatment
Coakley et al., 2022 [30]	Poor metabolic control: associated with higher BMI, greater body dissatisfaction, and increased psychological difficulties, including low mood, reduced emotional regulation, and impaired cognition	n/r
Haitjema et al., 2022 [38]	Age: mealtime behaviours like crying and tantrums were more common in younger children Eating environment: eating separate from the rest of the family may negatively affect appetite and feeding Strict routine of feeding and having to take PS: more difficulty with trying new foods	Increasing awareness among PKU health care workers about disordered eating Education of PKU families to minimise social restrictions
Rice et al., 2023 [46]	Delayed introduction of second stage PS (> 8 m of age): may decrease appetite for solid foods and contribute to long-term bottle use, limit exposure to spoon-fed textures and delay developmentally appropriate feeding skills	Assignment of a consistent lead dietitian to guide parents through each stage of complementary feeding Collaboration with multidisciplinary team (e.g., psychologists) strengthens parental support Timely transition to a second stage PS (ideally upon commencement of complementary feeding): supports development of spoon-feeding skills, reduces reliance on bottle feeding, and acceptance of textures
Rocha et al., 2023 [47]	Presence of gastrointestinal symptoms (nausea, vomiting, diarrhoea, reflux, constipation, stomach pains)	n/r
Viau et al., 2023 [48]	Partial response to drug (pegvaliase) treatment: may experience limited dietary liberalisation, increasing frustration around mealtimes Adapting to an unrestricted diet after pegvaliase: challenges include reluctance to accept high-protein foods after years of avoidance, unfamiliarity with preparing or cooking protein-rich meals, and the financial impact of purchasing foods.	Pegvaliase treatment (responders): Improved eating behaviour: reduced food neophobia, greater enjoyment and appreciation of food Improved mental health: better mood, emotional regulation, and cognitive clarity Greater satiety: increased intake of intact protein, supporting more typical appetite patterns Enhanced social aspects of eating: minimal dietary protein restrictions, shared meals, reduced feelings of difference, and more spontaneous and flexible food choices
Pinto et al., 2025 [49]	Gender: females demonstrated greater neophobic tendencies (heightened avoidance behaviours in unfamiliar dietary and social contexts)	n/r
Yilmaz et al., 2025 [50]	Lower maternal education: reduced access to nutritional knowledge, feeding strategies, and confidence in managing PKU Not attending nursery: limited exposure to structured mealtime routines, peer modelling, and external feeding support can hinder development of independent feeding skills Personal and family circumstances: single parenthood, parental unemployment, parental divorce, and having siblings with PKU can increase household stress and reduce capacity to maintain consistent feeding routines Co-existing illnesses: recurrent or chronic illness can disrupt feeding routines, reduce appetite, and increase food refusal	Higher maternal education: greater nutritional knowledge, confidence in feeding strategies, and familiarity with PKU management Attending full-time nursery: a structured environment with predictable routines, consistent expectations, and support from nursery staff
Yilmaz Nas et al., 2026 [51]	n/r	BH4 treatment (responsive): less neophobia
Albers et al., 2026 [52]	n/r	n/r
Giret et al., 2026 [53]	Classic PKU: more likely to experience eating disorders Psychiatric comorbidities: depressive episodes more common in classic PKU	n/r

Abbreviations: PKU: phenylketonuria; PS: protein substitute; SLPF: special low protein foods; BH4: sapropterin; n/r: not reported; OCD: obsessive–compulsive disorder; DM: diabetes mellitus; Phe: phenylalanine; m: months; BMI: body mass index. * World Health Organisation (WHO) advocate weaning from 6 months, but in PKU, it is considered beneficial to introduce solids from 4 months, as earlier exposure to foods has been shown to improve acceptance of a wider food variety, vital when diet is already severely limited with reliance on SLPFs.

## Data Availability

The original contributions presented in this study are included in the article/Supplementary Material. Further inquiries can be directed to the corresponding author.

## Supplementary Materials

The following supporting information can be downloaded at https://www.mdpi.com/article/10.3390/nu18152576/s1. Table S1: Search Strategy: Systematic Review of Eating Disorders and Disordered Eating in PKU; Table S2: Other data that may be related to disordered eating and feeding problems in patients with PKU.
