# Supplementary material for: Is Phenylalanine Restricted Dietary Treatment in Phenylketonuria Associated with Disordered Eating: A Systematic Review
Source: Nutrients. 2026 Aug 6;18(15):2576. doi: 10.3390/nu18152576 (PMC13467740; doi:10.3390/nu18152576)
Supplement: Supplementary file 1 [file nutrients-18-02576-s001.zip › Table S1_Search Strategy_SR_EatingDisPKU_25052026.pdf]

**Table S1.** Search Strategy: Systematic Review of Eating Disorders and Disordered Eating in PKU

**1. PUBMED**

#1: phenylketonuri*[Title/Abstract] OR PKU[Title/Abstract] OR "phenylalanine def*"[Title/Abstract] OR hyperphenylalaninemi*[Title/Abstract] OR "phenylalanine hydroxylase def*"[Title/Abstract] OR "PAH def*"[Title/Abstract]

#2: anorexi*[Title/Abstract] OR bulimi*[Title/Abstract] OR "binge eating"[Title/Abstract] OR "binge-eating"[Title/Abstract] OR neophobi*[Title/Abstract] OR "food phobia"[Title/Abstract] OR orthorexia*[Title/Abstract] OR "eating disorder*"[Title/Abstract] OR "disorder* eating"[Title/Abstract] OR "restrictive eating"[Title/Abstract] OR "eating restricti*"[Title/Abstract] OR "eating avoid*"[Title/Abstract] OR "avoidant eating"[Title/Abstract] OR "eating problem*"[Title/Abstract] OR "problem* eating"[Title/Abstract] OR "eating habit*"[Title/Abstract] OR "eating behavio*"[Title/Abstract] OR "eating attitude*"[Title/Abstract] OR "eating difficult*"[Title/Abstract] OR "food intake disorder"[Title/Abstract] OR "food disorder"[Title/Abstract] OR "restrictive food intake"[Title/Abstract] OR "food restricti*"[Title/Abstract] OR "food avoid*"[Title/Abstract] OR "avoidant restrictive food intake"[Title/Abstract] OR "food refusal"[Title/Abstract] OR "food aversion"[Title/Abstract] OR "food habit*"[Title/Abstract] OR "food choice*"[Title/Abstract] OR "food intake behavio*"[Title/Abstract] OR "food acceptance"[Title/Abstract] OR "food prefer*"[Title/Abstract] OR "feeding disorder*"[Title/Abstract] OR "disorder* feeding"[Title/Abstract] OR "feeding restricti*"[Title/Abstract] OR "restrictive feeding"[Title/Abstract] OR "feeding refusal"[Title/Abstract] OR "feeding aversion"[Title/Abstract] OR "feeding problem"[Title/Abstract] OR "feeding habit*"[Title/Abstract] OR "feeding behavio*"[Title/Abstract] OR "feeding attitude*"[Title/Abstract] OR "feeding difficult*"[Title/Abstract] OR "poor appetite"[Title/Abstract] OR obesity[Title/Abstract] OR "taste aversion"[Title/Abstract] OR "oral aversion"[Title/Abstract] OR "food texture"[Title/Abstract] OR "food fad*"[Title/Abstract] OR "food preoccupation"[Title/Abstract] OR "body image"[Title/Abstract] OR "irregular eating"[Title/Abstract] OR "eating patterns"[Title/Abstract] OR "irregular meals"[Title/Abstract] OR "meal patterns"[Title/Abstract] OR "diet* pattern*"[Title/Abstract] OR "control* eating"[Title/Abstract] OR "diet* disorder"[Title/Abstract] OR "diet* habit*"[Title/Abstract] OR "diet* behavio*"[Title/Abstract] OR "food avoidance"[Title/Abstract] OR "skip* meal*"[Title/Abstract] OR "skip* breakfast"[Title/Abstract] OR "laxative abuse"[Title/Abstract] OR "alcohol abuse"[Title/Abstract] OR "alcohol addiction"[Title/Abstract] OR "alcohol disorder"[Title/Abstract] OR "alcohol misuse"[Title/Abstract] OR "drug addiction"[Title/Abstract] OR "self-harm"[Title/Abstract] OR "self harm"[Title/Abstract] OR "atypical eating"[Title/Abstract]

#3: #1 AND #2

**2. SCOPUS**

#1: TITLE-ABS-KEY ( phenylketonuri* OR pku OR "phenylalanine def*" OR hyperphenylalaninemi* OR "phenylalanine hydroxylase def*" OR "pah def*" )

#2: TITLE-ABS-KEY ( anorexi* OR bulimi* OR "binge eating" OR "binge-eating" OR neophobi* OR "food phobia" OR orthorexia* OR "eating disorder*" OR "disorder* eating" OR "restrictive eating" OR "eating restricti*" OR "eating avoid*" OR "avoidant eating" OR "eating problem*" OR "problem* eating" OR "eating habit*" OR "eating behavio*" OR "eating attitude*" OR "eating difficult*" OR "food intake disorder" OR "food disorder" OR "restrictive food intake" OR "food restricti*" OR "food avoid*" OR "avoidant restrictive food intake" OR "food refusal" OR "food aversion" OR "food habit*" OR "food choice*" OR "food intake behavio*" OR "food acceptance" OR "food prefer*" OR "feeding disorder*" OR "disorder* feeding" OR "feeding restricti*" OR "restrictive feeding" OR "feeding refusal" OR "feeding aversion" OR "feeding problem" OR "feeding habit*" OR "feeding behavio*" OR "feeding attitude*" OR "feeding difficult*" OR "poor appetite" OR obesity OR "taste aversion" OR "oral aversion" OR "food texture" OR "food fad*" OR "food preoccupation" OR "body image" OR "irregular eating" OR "eating patterns" OR "irregular

| |
|---|
| meals" OR "meal patterns" OR "diet* pattern*" OR "control* eating" OR "diet* disorder" OR "diet* habit*" OR "diet* behavio*" OR "food avoidance" OR "skip* meal*" OR "skip* breakfast" OR "laxative abuse" OR "alcohol abuse" OR "alcohol addiction" OR "alcohol disorder" OR "alcohol misuse" OR "drug addiction" OR "self-harm" OR "self harm" OR "atypical eating" )<br>#3: #1 AND #2 |
| **3. CENTRAL Cochrane Library** |
| #1: (phenylketonuri* OR PKU OR “phenylalanine def*” OR hyperphenylalaninemi* OR “phenylalanine hydroxylase def*” OR “PAH def*”):ti,ab,kw<br>#2: (anorexi* OR bulimi* OR "binge eating" OR "binge-eating" OR neophobi* OR “food phobia” OR orthorexia* OR "eating disorder*" OR "disorder* eating" OR "restrictive eating" OR "eating restricti*" OR "eating avoid*" OR "avoidant eating" OR "eating problem*" OR "problem* eating" OR "eating habit*" OR "eating behavio*" OR "eating attitude*" OR "eating difficult*" OR "food intake disorder" OR “food disorder” OR "restrictive food intake" OR "food restricti*" OR "food avoid*" OR "avoidant restrictive food intake" OR "food refusal" OR "food aversion" OR "food habit*" OR "food choice*" OR "food intake behavio*" OR "food acceptance" OR “food prefer*” OR "feeding disorder*" OR "disorder* feeding" OR "feeding restricti*" OR "restrictive feeding" OR "feeding refusal" OR "feeding aversion" OR "feeding problem" OR "feeding habit*" OR "feeding behavio*" OR "feeding attitude*" OR "feeding difficult*" OR “poor appetite” OR obesity OR “taste aversion” OR “oral aversion” OR “food texture” OR “food fad*” OR “food preoccupation” OR “body image” OR “irregular eating” OR “eating patterns” OR “irregular meals” OR “meal patterns” OR “diet* pattern*” OR “control* eating” OR “diet* disorder” OR “diet* habit*” OR “diet* behavio*” OR “food avoidance” OR “skip* meal*” OR “skip* breakfast” OR “laxative abuse” OR “alcohol abuse” OR “alcohol addiction” OR “alcohol disorder” OR “alcohol misuse” OR “drug addiction” OR “self-harm” OR “self harm” OR “atypical eating”):ti,ab,kw<br>#3: #1 AND #2 |
| **4. WEB OF SCIENCE** |
| #1: TS=(phenylketonuri* OR PKU OR “phenylalanine def*” OR hyperphenylalaninemi* OR “phenylalanine hydroxylase def*” OR “PAH def*”)<br>#2: TS=(anorexi* OR bulimi* OR "binge eating" OR "binge-eating" OR neophobi* OR “food phobia” OR orthorexia* OR "eating disorder*" OR "disorder* eating" OR "restrictive eating" OR "eating restricti*" OR "eating avoid*" OR "avoidant eating" OR "eating problem*" OR "problem* eating" OR "eating habit*" OR "eating behavio*" OR "eating attitude*" OR "eating difficult*" OR "food intake disorder" OR “food disorder” OR "restrictive food intake" OR "food restricti*" OR "food avoid*" OR "avoidant restrictive food intake" OR "food refusal" OR "food aversion" OR "food habit*" OR "food choice*" OR "food intake behavio*" OR "food acceptance" OR “food prefer*” OR "feeding disorder*" OR "disorder* feeding" OR "feeding restricti*" OR "restrictive feeding" OR "feeding refusal" OR "feeding aversion" OR "feeding problem" OR "feeding habit*" OR "feeding behavio*" OR "feeding attitude*" OR "feeding difficult*" OR “poor appetite” OR obesity OR “taste aversion” OR “oral aversion” OR “food texture” OR “food fad*” OR “food preoccupation” OR “body image” OR “irregular eating” OR “eating patterns” OR “irregular meals” OR “meal patterns” OR “diet* pattern*” OR “control* eating” OR “diet* disorder” OR “diet* habit*” OR “diet* behavio*” OR “food avoidance” OR “skip* meal*” OR “skip* breakfast” OR “laxative abuse” OR “alcohol abuse” OR “alcohol addiction” OR “alcohol disorder” OR “alcohol misuse” OR “drug addiction” OR “self-harm” OR “self harm” OR “atypical eating”)<br>#3: #1 AND #2 |
