# Supplementary material for: Is Phenylalanine Restricted Dietary Treatment in Phenylketonuria Associated with Disordered Eating: A Systematic Review
Source: Nutrients. 2026 Aug 6;18(15):2576. doi: 10.3390/nu18152576 (PMC13467740; doi:10.3390/nu18152576)
Supplement: Supplementary file 1 [file nutrients-18-02576-s001.zip › Table S2_Anthropometric and metabolic control data.pdf]

Table S2. Other data that may be related to disordered eating and feeding problems in patients with PKU.

Reference	Sample (population vs. comparator)	Outcomes	Method of assessment	Key findings
				Underweight, Overweight and Obesity
Bentovim et al., 1970 [41]	Children with PKU:<br>Group A (n=6):<br>Hydrolysate PS<br>vs.<br>Group B (n=6):<br>L-AA PS	• Underweight, overweight and obesity	• BMI for age	• Underweight: None<br>• Overweight: None<br>• Obesity: Group A: None; Group B: 1/6 (17%); Total sample: 1/12 (8%)
Thiele et al., 2015 [42]	Children with PKU (n=8):<br>Pre-BH4 (diet only; n=8)<br>vs.<br>Post-BH4 (n=5; fully liberalized diet; n=3 partially liberalized diet)<br>vs.<br>Healthy population	• Underweight, overweight and obesity	• BMI for age	**Pre-BH4:**<br>• Underweight: 1/8 (13%)<br>• Overweight: 1/8 (13%)<br>• Obesity: None<br><br>**Post-BH4:**<br>• Underweight: None<br>• Overweight: None<br>• Obesity: None<br><br>**Healthy controls**: n/r
Evans et al., 2017 [44]	Infants with PKU (n=31)	• Underweight, overweight and obesity	• Weight and BMI for age	• Underweight: 3/31 (10%) at 6 and 12 m; none at 24 m<br>• Overweight: None<br>• Obesity: None
Evans et al., 2019a [36] and Evans et al., 2019b [33]	Infants with PKU (n=20)<br>vs.<br>Healthy controls (n=20)	• Underweight, overweight and obesity	• BMI for age	• Underweight:<br>Infants with PKU: None<br>(Healthy controls: 1/20 (5%) at 12 and 24 m)<br>• Overweight:<br>Infants with PKU: 1/20 (5%) at 12 and 24 m<br>(Healthy controls: 2/20 (10%) at 12 and 24 m)<br>• Obesity: None

Table S2. Other data that may be related to disordered eating and feeding problems in patients with PKU.

Reference	Sample (population vs. comparator)	Outcomes	Method of assessment	Key findings
Luu et al., 2020 [40]	Adolescents and adults with PKU (n=15; n=3 not on restricted diet)	• Underweight, overweight and obesity	• BMI	• Underweight: None<br>• Overweight: None<br>• Obesity: None
Viau et al., 2021 [45]	Adults with PKU on pegvaliase treatment (n=18)	• Underweight, overweight and obesity	• BMI	• Underweight: None<br>• Overweight: 9/18 (50%)<br>• Obesity: 7/18 (39%)
Coakley et al., 2022 [30]	Adults with PKU (n=41) vs. Healthy controls (n=41)	• Underweight, overweight and obesity	• BMI	• Underweight: None<br>• Overweight: 13/41 (32%)<br>• Obesity: 14/41 (34%)<br>• Mean BMI: PKU 30.1; controls 25.9kg/m^2
Viau et al., 2023 [48]	Adults with PKU on pegvaliase treatment (n=11)	• Underweight, overweight and obesity	• BMI	• Underweight: None<br>• Overweight: 6/11 (55%)<br>• Obesity: 4/11 (36%)
Pinto et al., 2025 [49]	Adolescents with PKU on diet only (n=30) and on diet + BH4 treatment (n=3)	• Underweight, overweight and obesity	• BMI for age	• Underweight: None<br>• Overweight: 6/33 (18%) (Males: 4/25; 25% vs. Females: 4/17; 24%)<br>• Obesity: 7/33 (21%) (Males: 2/16; 13% vs. Females: 5/17; 29%)
Yilmaz et al., 2025 [50]	Children with PKU (n=12)	• Underweight, overweight and obesity	• Weight and BMI for age	• Underweight: None<br>• Overweight: None<br>• Obesity: None
Albers et al., 2026 [52]	Adults with PKU on diet (n=36), on BH4 treatment (n=3) and on pegvaliase treatments (n=2)	• Underweight, overweight and obesity	• BMI	• Underweight: 2/41 (5%)<br>• Overweight: None<br>• Obesity: 10/41 (24%)

Table S2. Other data that may be related to disordered eating and feeding problems in patients with PKU.

Reference	Sample (population vs. comparator)	Outcomes	Method of assessment	Key findings
Metabolic control				
Bentovim et al., 1970 [41]	Children with PKU:<br>Group A (n=6):<br>Hydrolysate PS<br>vs.<br>Group B (n=6):<br>L-AA PS	• Blood Phe	• Laboratory DBS µmol/L<br>• Target range: 120-240	• Group A mean ± SD; median[range]: 253.4 ± 126; 229.2 [132-474]<br>• Group B mean ± SD; median[range]: 345 ± 91; 336 [240-469]<br>• Total sample mean ± SD; median[range]: 299.2 ± 115.5; 283.2 [132-474]
Thiele et al., 2015 [42]	Children with PKU (n=8):<br>Pre-BH4 (diet only; n=8)<br>vs.<br>Post-BH4 (n=5; fully liberalized diet; n=3 partially liberalized diet)<br>vs.<br>Healthy population	• Blood Phe	• Laboratory DBS µmol/L<br>• Target range: 120-360 (≤12y); 120-600 (>12y)	• Pre-BH4 (diet only) mean ± SD: 262.2±129.4; mean % above target: 13.2±23.7<br>• 1 year on BH4 + diet mean ± SD: 337.1±129.6; mean % above target: 13.2±23.7<br>• 2 years on BH4 + diet mean ± SD: 382.7±148.1; mean % above target: 14.3±28.1<br>• 3 years on BH4 + diet mean ± SD: 371.7±119.8; mean % above target: 15.1±28.1
Roberts et al., 2017 [43]	Adults with PKU on diet treatment (n=7), off diet (n=1)	• Blood Phe	• Laboratory µmol/L (method n/r)<br>• Target range: n/r	• Phe levels in target range (% over lifetime):<br>n=6 100% of the time<br>n=1 80% of the time<br>n=1 for <10% of the time
Evans et al., 2017 [44]	Infants with PKU (n=31)	• Blood Phe	• Laboratory DBS µmol/L<br>• Target range 120-360	• Median (range) weaning to 12m of age: 175 (990-310)<br>• Median (range) 12-24m of age: 180 (100-400)
Evans et al., 2019a [36] and Evans et al., 2019b [33]	Infants with PKU (n=20)<br>vs.<br>Healthy controls (n=20)	• Blood Phe	• Laboratory DBS µmol/L<br>• Target range 12--360	**Mean blood Phe by age (range):**<br>• 3m: 160 (87-246) - 14m: 260 (45-595)<br>• 4m: 177 (40-340) - 15m: 240 (143-500)<br>• 5m: 202 (120-334) - 16m: 272 (114-707)<br>• 6m: 200 (117-357) - 17m: 255 (110-567)<br>• 7m: 183 (70-405) - 18m: 296 (118-753)

Table S2. Other data that may be related to disordered eating and feeding problems in patients with PKU.

Reference	Sample (population vs. comparator)	Outcomes	Method of assessment	Key findings
				• 8m: 203 (70-453) - 19m: 240 (87-470)<br>• 9m: 201 (25-408) - 20m: 227 (68-576)<br>• 10m: 227 (73-632) - 21m: 233 (98-410)<br>• 11m: 250 (104-547) - 22m: 237 (97-462)<br>• 12m: 221 (64-362) - 23m: 212 (97-462)<br>• 13m: 275 (53-854) - 24m: 240 (110-402)
Luu et al., 2020 [40]	Adolescents and adults with PKU (n=15; n=3 not on restricted diet)	• Blood Phe	• Laboratory µmol/L (method n/r)<br>• Target range: 120-360	• 66% (n=10) between 120-360umol/L[a]<br>• 20% (n=3) 360-600umol/L[a]<br>• 13% (n=2) 600-1200umol/L[a]
Viau et al., 2021 [45]	Adults with PKU on pegvaliase treatment (n=18)	• Blood Phe<br>• Blood tyrosine	• Laboratory DBS and venous blood (pre study) and fasting venous blood (during study) µmol/L<br>• Blood Phe target range: 30-360<br>• Blood tyrosine target range: >30	**Blood Phe 6m prior to enrolment median (IQR)**:<br>• 151 (2–257); n=5 <30<br>**Blood Phe during study median (IQR):**<br>• 12 (1–339); n=11 <30; n=3 >600<br>**Blood tyrosine during study median (IQR):**<br>• 33 (31–39); n=10, slightly low (<34)
Coakley et al., 2022 [30]	Adults with PKU (n=41)<br>vs.<br>Healthy controls (n=41)	• Blood Phe	• Laboratory µmol/L (method n/r)<br>• Target range: n/r	• Mean: 381
Rice et al., 2023 [46]	Infants with PKU of diet treatment (n=21)	• Blood Phe	• Laboratory µmol/L (method n/r)<br>• Target range: n/r	• Breastfed mean ± SD: 335±54<br>• Formula fed mean ± SD: 357±52
Viau et al., 2023 [48]	Adults with PKU on pegvaliase treatment (n=11)	• Blood Phe<br>• Blood tyrosine	• Laboratory fasting venous blood µmol/L<br>• Blood Phe target range: 30-360<br>• Blood tyrosine target range: >30	**Blood Phe baseline mean ± SD:**<br>• Responders (n=6): 547 ± 324<br>• Partial responders (n=5): 1140 ± 218<br>• n=5 had one or more low blood Phe<br>**Blood Phe median % (IQR) change from baseline to 15m:**<br>• Responders (n=6): 95% (-98 to -81)<br>• Partial responders (n=5): 18% (-39 to 16)<br>**Blood tyrosine:**

Table S2. Other data that may be related to disordered eating and feeding problems in patients with PKU.

Reference	Sample (population vs. comparator)	Outcomes	Method of assessment	Key findings
				• n=6 had one or more low blood tyrosine
Yilmaz et al., 2025 [50]	Children with PKU (n=12)	• Blood Phe	• Laboratory DBS µmol/L<br>• Target range: 120-360	**6m pre-baseline median (range):**<br>• Smooth transition: 180 (140-260) (n=5)<br>• Transition with difficulty: 470 (330-665) (n=3)<br>• Transition failure: 170 (120-290) (n=4)<br>**During transition median (range):**<br>• Smooth transition: 200 (160-310) (n=5)<br>• Transition with difficulty: 430 (310-750) (n=3)<br>• Transition failure: 200 (120-340) (n=4)<br>**6m post transition median (range):**<br>• Smooth transition: 210 (160-320) (n=5)<br>• Transition with difficulty: 550 (400-750) (n=3)<br>• Transition failure: 200 (140-388) (n=4)
Pinto et al., 2025 [49]	Adolescents with PKU on diet only (n=30) and on diet + BH4 treatment (n=3)	• Blood Phe	• Laboratory DBS µmol/L<br>• Target range: 120-600	**Over 1 year mean ± SD:**<br>• 405 ± 105<br>• Males: 473 ± 226; Females: 341 ± 107<br>**% of blood Phe <600 mmol/L mean ± SD:**<br>• 83 ± 30%<br>• Males: 73 ± 39%; Females: 94 ± 8%<br>**% of blood Phe <360 mmol/L mean ± SD:**<br>• 49 ± 33%<br>• Males: 41 ± 35%; Females: 56 ± 29%
Yilmaz et al., 2026 [51]	Children with PKU diet treatment only (n=12)<br>vs<br>Diet + BH4 treatment (n=21)	• Blood Phe	• Laboratory DBS µmol/L<br>• Target range: 120-360 (≤12y); 120-600 (>12y)	**Diet only <12y (n=6) median (Q1-Q3); % above target:**<br>• pre-baseline: 300 (200-400); 30%<br>• 0-6m: 295 (178-460); 37%<br>• 6-12m: 310 (200-400); 29%<br>• 12-24m: 285 (188-420); 32%<br>**Diet only ≥12y (n=6) median (Q1-Q3); % above target:**<br>• pre-baseline: 410 (220-520); 10%<br>• 0-6m: 435 (320-570); 14%<br>• 6-12m: 540 (420-640); 30%<br>• 12-24m: 600 (470-750); 47%

Table S2. Other data that may be related to disordered eating and feeding problems in patients with PKU.

Reference	Sample (population vs. comparator)	Outcomes	Method of assessment	Key findings
				**Diet + BH4 <12y (n=13) median (Q1-Q3); % above target:**<br>• pre-baseline: 270 (210-330); 16%<br>• 0-6m: 240 (200-310); 12%<br>• 6-12m: 240 (180-310); 11%<br>• 12-24m: 250 (190-320); 16%<br>**Diet + BH4 ≥12y (n=8) median (Q1-Q3); % above target:**<br>• pre-baseline: 420 (280-550); 14%<br>• 0-6m: 400 (240-590); 19%<br>• 6-12m: 370 (205-595); 21%<br>• 12-24m: 455 (233-638); 27%
Albers et al., 2026 [52]	Adults with PKU on diet (n=36), on BH4 treatment (n=3) and on pegvaliase treatments (n=2)	• Blood Phe	• Laboratory DBS and venous blood µmol/L<br>• Target range: 120-600	**Over study period mean ± SD:**<br>• 998.3 ± 290.4<br>**Initial appointment mean ± SD; median:**<br>• 988.6 ± 469.5; 780 (n = 44)<br>**First annual f/up mean ± SD:**<br>• 1070.2 ± 396.9; 780 (n = 28)<br>**2nd annual f/up mean ± SD:**<br>• 1073.3 ± 421.5; 900 (n = 19)<br>**3rd annual f/up mean ± SD:**<br>• 805.9 ± 392.6; 900 (n = 13)<br>**% of Phe within target range:**<br>• 38% (n=43) between initial and 1st f/up<br>• 31% (n=27) between 1st & 2nd f/up<br>• 20% (n=20) between 2nd & 3rd f/up<br>**Highest %n in range:**<br>• 50% (n=12) after 3rd f/up
Giret et al., 2026 [53]	n=187 adults with PKU: on diet (n=104), off diet (n=83), on BH4 treatment (n=5)[b]	• Blood Phe	• Laboratory µmol/L (method n/r)<br>• Target range: n/r	**At study start median (IQR):**<br>• ALL: 1050 (709-1410) n=145[c]<br>• cPKU: 1156 (833-1488) n=115[c]<br>• mPKU: 730 (636-914) n=23[c]<br>• mHPA: 321 (156-419) n=7[c]

Abbreviations: PKU: Phenylketonuria; PS: protein substitute; L-AA PS: L-amino acid-based protein substitute; BMI: body mass index; BH4: sapropterin; m: months; y: years; DBS: dried blood spot; SD: standard deviation; cPKU: classic phenylketonuria; mPKU: mild phenylketonuria; mHPA: mild hyperphenylalaninemia; F/up: follow up

[a] Not clear which ones were on a restricted diet; not clear if mean or median values

[b] Not clear if patients on BH4 were on diet also

[c] Unclear if these included patients "on" or "off diet" or on BH4 treatment; however, the authors report that an analysis of the prevalence of psychiatric disorders in patients "on diet" and "off diet" did not find any statistically significant difference (p=0.11)
